# Understanding immune system dysfunction and its context in mood disorders: psychoneuroimmunoendocrinology and clinical interventions

**DOI:** 10.1186/s40779-024-00577-w

**Published:** 2024-12-17

**Authors:** Miguel A. Ortega, Oscar Fraile-Martinez, Cielo García-Montero, Raul Diaz-Pedrero, Laura Lopez-Gonzalez, Jorge Monserrat, Silvestra Barrena-Blázquez, Miguel Angel Alvarez-Mon, Guillermo Lahera, Melchor Alvarez-Mon

**Affiliations:** 1 Department of Medicine and Medical Specialities, Faculty of Medicine and Health Sciences, University of Alcalá, 28801 Alcalá de Henares, Spain; 2 Ramón y Cajal Institute of Sanitary Research IRYCIS, 28034 Madrid, Spain; 3 Department of Surgery, Medical and Social Sciences, Faculty of Medicine and Health Sciences, University of Alcalá, 28801 Alcala de Henares, Spain; 4 Department of Nursing and Physiotherapy, Faculty of Medicine and Health Sciences, University of Alcalá, 28801 Alcalá de Henares, Spain; 5 Department of Psychiatry and Mental Health, Hospital Universitario Infanta Leonor, 28031 Madrid, Spain; 6 Psychiatry Service, Center for Biomedical Research in the Mental Health Network, University Hospital Príncipe de Asturias, 28806 Alcalá de Henares, Spain; 7 Immune System Diseases-Rheumatology and Internal Medicine Service, University Hospital Príncipe de Asturias, CIBEREHD, 28806 Alcalá de Henares, Spain

**Keywords:** Mood disorders, Immune system, Neuroinflammation, Systemic inflammation, Psychoneuroimmunoendocrinology (PNIE), Pharmacological interventions, Lifestyle medicine

## Abstract

Mood disorders include a set of psychiatric manifestations of increasing prevalence in our society, being mainly represented by major depressive disorder (MDD) and bipolar disorder (BD). The etiopathogenesis of mood disorders is extremely complex, with a wide spectrum of biological, psychological, and sociocultural factors being responsible for their appearance and development. In this sense, immune system dysfunction represents a key mechanism in the onset and pathophysiology of mood disorders, worsening mainly the central nervous system (neuroinflammation) and the periphery of the body (systemic inflammation). However, these alterations cannot be understood separately, but as part of a complex picture in which different factors and systems interact with each other. Psychoneuroimmunoendocrinology (PNIE) is the area responsible for studying the relationship between these elements and the impact of mind–body integration, placing the immune system as part of a whole. Thus, the dysfunction of the immune system is capable of influencing and activating different mechanisms that promote disruption of the psyche, damage to the nervous system, alterations to the endocrine and metabolic systems, and disruption of the microbiota and intestinal ecosystem, as well as of other organs and, in turn, all these mechanisms are responsible for inducing and enhancing the immune dysfunction. Similarly, the clinical approach to these patients is usually multidisciplinary, and the therapeutic arsenal includes different pharmacological (for example, antidepressants, antipsychotics, and lithium) and non-pharmacological (i.e., psychotherapy, lifestyle, and electroconvulsive therapy) treatments. These interventions also modulate the immune system and other elements of the PNIE in these patients, which may be interesting to understand the therapeutic success or failure of these approaches. In this sense, this review aims to delve into the relationship between immune dysfunction and mood disorders and their integration in the complex context of PNIE. Likewise, an attempt will be made to explore the effects on the immune system of different strategies available in the clinical approach to these patients, in order to identify the mechanisms described and their possible uses as biomarkers.

## Background

Affective or mood disorders comprise a continuum of components of depression and mania, appearing alone or in combination [1]. It is estimated that approximately 1 in 4 individuals will suffer at least once in their life from a type of affective disorder, that is highly disabling for the subject who suffers from it [2]. Globally, affective disorders mainly include major depressive disorder (MDD), type I, and type II bipolar disorders (BDs). Similarly, other types of conditions are considered such as cyclothymic disorder, seasonal affective disorder (SAD), premenstrual dysphoric disorder (PMDD), persistent depressive disorder or dysthymia, disruptive mood dysregulation disorder, and bipolar/depressive induced by drugs or comorbidities [3]. The diagnosis of affective disorders follows the criteria included in the clinical guide par excellence in psychiatry, the Diagnostic and Statistical Manual of Mental Disorders, Fifth Edition (DSM-5), although there are other reference manuals such as the International Classification of Diseases‑11, with similarities and some differences in the diagnostic criteria [3–5]. However, due to the lack of objective tests and the perceived presence of social stigma, mood disorders are frequently underdiagnosed or misdiagnosed, which adds to the fact that a significant percentage may be resistant to the treatment received [2]. Likewise, there is relatively little knowledge of the etiopathogenic processes involved in affective disorders, so it is necessary to delve into the different mechanisms that are currently described in the literature [6].

In this context, the biopsychosocial model defends the existence of interrelated social, biological, and psychological determinants that help to understand the origin of health and disease [7]. In this sense, the relevance of the interconnection of multiple sociocultural, psychological, and biological factors in the onset and development of affective disorders has been described [8–10]. Regarding the biology of affective disorders, the existence of numerous molecular, cellular, structural, and functional changes in various regions of the brain and the central nervous system (CNS) has been demonstrated, aiding to understand the manifestations of these patients [11]. Conversely, affective disorders are also accompanied by changes and disruptions at the systemic level, so the changes that occur in the weakened organism affect the brain and vice versa [12, 13]. In this sense, different fields of study such as psychoimmunology or affective immunology have shown how there is a close interrelationship between the immune system and mood [14]. This immune system-psyche communication occurs in a bidirectional way and it is even more correct to understand this connection in the context of psychoneuroimmunoendocrinology (PNIE), where the psyche, nervous, immune, and endocrine systems compose a single unit that acts interdependently [15, 16]. In health conditions, there is an optimal balance between the different elements of the PNIE. However, in affective disorders, there is a clear disruption of these components, with the immune system being especially affected in these subjects [17]. Indeed, there is increasing evidence about the key role that inflammation plays in the pathophysiology and the course of affective disorders, being suggested as a treatment target to consider in these patients [18–20].

In this panorama, the objective of this review is to collect and transmit the main knowledge found in the available scientific literature about the immunopathogenic mechanisms of the main affective disorders. In the same way, an attempt will be made to address different strategies and approaches aimed at modulating the immunoinflammatory system of these patients, to create a global vision of the importance of the immune system as a therapeutic target or offering potential biomarkers to be considered in the comprehensive treatment of patients with affective disorders.

## The role of immunoinflammatory dysfunction in affective disorders

The immune system has a close relationship with the different systems of the organism, including the brain, affecting its functioning in conditions of health and disease [21]. Alterations in the immune system are a potential trigger for the psychosomatic manifestations of affective disorders, as well as their pathophysiology and etiopathogenesis [22]. It is difficult to establish whether changes in the immune-inflammatory system are a cause or a consequence of the known pathophysiological events that underlie affective disorders. For example, in the case of MDD or BD, the importance of inflammation and changes in responses mediated by the innate and adaptive immune system as a key modifier of this type of disorder is noted, although it is conceived that these changes are interrelated with the different biopsychosocial events that accompany these conditions [23, 24]. In this sense, it is recognized that the immune system can promote and aggravate the development of affective disorders from two main pathways: inflammation in the CNS (neuroinflammation), and systemic inflammation [13, 25]. In turn, a significant number of elements for example psychological stress, neuroendocrine disruptions themselves, metabolic and endocrine changes, intestinal dysbiosis, and multiple lifestyle factors such as malnutrition or a sedentary lifestyle promote an exacerbated inflammatory response and interact in a bidirectional manner with the immune system, being also involved in the development of affective disorders [26]. In this section, we will first summarize how inflammation affects the development of affective disorders to later integrate and understand its relationship with the other factors associated with these psychiatric conditions.

### Neuroinflammation

Firstly, neuroinflammation is a common point of the various neuropsychiatric pathologies of acute or chronic origin [27]. It is important to highlight that transient low- or medium-grade neuroinflammatory processes can promote processes of neurodevelopment, neuroprotection, or tissue repair; however, if the stimuli that promote neuroinflammation are prolonged and become chronic over time or intensify, they can lead to neuronal damage mechanisms, cognitive dysfunctions, or the appearance of affective disorders [28]. In the case of the latter, it is known that neuroinflammatory mechanisms promote a series of pathophysiological events involved in these diseases, including the disruption of neurotransmitters in the brain, alterations in different neuroendocrine axes, mechanisms of cell damage, and changes in neurogenesis and neuroplasticity of the brain [26]. A large part of these effects is due to the action of a series of pro-inflammatory cytokines, among which interleukin (IL)-1β, IL-6, and tumor necrosis factor-α (TNF-α) stand out. These cytokines critically modulate neural circuits and glial cells in the CNS, promoting damage and neuroinflammation-mediated disturbances in patients with affective disorders [29]. Many of these pro-inflammatory cytokines induce what is called “sickness behavior”, which consists of changes in motivational centers aimed at reorganizing the body’s priorities to deal with the threat [30]. In acute conditions, this “sickness behavior” is mostly beneficial, but when it becomes chronic, it will promote and contribute to the appearance and development of affective disorders, especially in MDD [31]. This mechanism modulated by cytokines acts either through afferent neuronal pathways or crossing the so-called blood–brain barrier (BBB). The BBB is a fundamental structure made up of endothelial cells, their basement membrane, and perivascular astrocytes that tightly regulate what type of substances pass from the body to the CNS [32]. Recent hypotheses defend those patients with affective disorders present a transient or permanent disruption of the integrity of the BBB, as well as an increase in its permeability, allowing the entry of inflammatory mediators and immune cells from peripheral blood into the CNS [33–35]. These events are preceded by the release of damage-associated molecular patterns (DAMPs) or pathogen-associated molecular patterns (PAMPs) recognized by pattern recognition receptors localized in the immune cells. In patients with neuropsychiatric disorders, hyperactivation of these receptors has been observed, as well as an increase in DAMPs and PAMPs that stimulate neuroinflammatory responses [26].

At the cellular level, microglia are the main modulators of the different neuroinflammation mechanisms that occur in the brain of patients with affective disorders and other psychopathologies [36]. More in detail, these cells are activated in the presence of certain inflammatory mediators, DAMPs or PAMPs, and are polarized in a pro-inflammatory (M1) phenotype, mainly involved in neuroinflammatory mechanisms or an anti-inflammatory phenotype (M2), more related to the repair and neuroprotective actions. Each phenotype is characterized by the expression of a different profile of cytokines and chemokines, although according to current scientific evidence, there is a wide heterogeneity regarding the profiles presented by microglia in affective disorders [37]. Therefore, the role of microglial cells in different neuropsychiatric pathologies and mood disorders still needs to be explored in greater depth. Similarly, both astrocytes and oligodendrocytes also play a central role in the neuroinflammatory response. For example, astrocytes fulfill numerous support functions in the CNS, being involved in the repair of injuries in the brain and the response to aggressions, having continuous interaction with microglia [38]. Likewise, as previously mentioned, astrocytes are part of the BBB, which determine the circulating molecules that will reach the CNS, thus playing an important role in the neuroinflammatory response. Oligodendrocytes, for their part, form the myelin sheath of neurons in the CNS, serving not only as a structural element but also as a key modulator of neuronal function, interacting closely with them in the paranodes, located near the nodes of Ranvier [39]. In addition, it is widely accepted that there is an infiltration of T and B cells in the CNS parenchyma under the neuroinflammatory environment in patients with affective disorders, demonstrated in a post-mortem study [40], being favored by the disruption of the BBB, neuronal damage, activation of microglia, and dysregulation of astrocytes and oligodendrocytes [41]. In addition to microglia, perivascular brain macrophages also represent an important group of cells derived from the monocyte lineage that play an inflammatory role in the CNS [32]. In more detail, these cells are responsible for the production of various pro-inflammatory cytokines as well as facilitating the passage of leukocytes through the BBB [42]. The dysregulation of aforementioned glial cells is due to the unbalance in the yet novel term “glymphatic system”, which was coined referring to the lymphatic functions aided by glial cells in the CNS [43]. This is a clearance system of waste and toxins, consisting in the flow of cerebrospinal fluid through the brain tissue, more concretely, water clearence between the perivascular space and the brain parenchyma. This transport is mainly mediated by channels of aquaporin-4 on the astrocytic endfeet [44]. The process has heightened activity during sleep, believed to play a vital role in eliminating waste metabolites like β-amyloid protein [45]. As a whole, the different populations of glial cells interact and modulate each other, with neurons and the immune system, thus determining the neuroinflammatory response that occurs in the CNS of patients with affective disorders.

As collected herein, the neuroinflammatory response represents a key etiopathogenic mechanism in affective disorders, which in turn feeds back with other pathophysiological mechanisms that occur in the CNS of these patients. To further understand the neuroinflammatory context in patients with affective disorders see section “Changes in the CNS and the context of neuroinflammation”.

### Systemic immune dysfunction

Affective disorders are also related to a profound immunological dysfunction affecting both the innate and adaptative immune systems. The innate immune system is the first line of defense of the body and appears represented by different immune myeloid cells (monocytes/macrophages, dendritic cells, neutrophils, eosinophils, and basophils) and lymphoid cells like natural killer (NK) cells [46]. These cells are activated after recognition of PAMPs and DAMPs, leading to the release of innate cytokines like those belonging to the IL-1β, IL-6, and TNF families to initiate the immune response against pathogens and injuries and recruit other immune cells [47]. Adaptative responses appear later than innate responses and are conducted by T and B lymphocytes. Adaptative immunity provides broader and highly specific responses after recognition for both self- and nonself-antigens by the action of antigen-presenting cells (APCs), mainly dendritic cells [48]. Lymphocytes are regarded as naive and inactive cells until they are stimulated by their specific antigen. Then, they become activated and undergo clonal differentiation to become fully functional effector, and a subset of these cells persist as memory or effector-memory populations for performing more rapid adaptative responses if the antigen is recognized again [49]. After activation, B cells divide clonally to become plasma cells, generating antibodies against these antigens. Three main types of T cells are recognized: cytotoxic T lymphocytes (CTLs; also named CD8 T cells), helper T cells (Th) and regulatory T cells (Tregs), both subtypes also identified as CD4 T cells [50]. CTLs finds and kills the cells expressing its specific antigen (i.e., infected or carcinogenic cells); Tregs decrease the inflammatory activity to prevent exacerbated immune responses and Th influence the behavior and activity of other immune cells polarizing into different subtypes like Th1, Th2, Th17, and more [51]. Also, adaptative immune cells release specific cytokines like interferon-γ (IFN-γ), IL-17, IL-4, or IL-5, aiding to perform the most adequate responses to each threat [47]. A growing body of evidence supports the relevance of innate and adaptive dysfunction in the development of mood disorders [14, 23, 52]. According to these studies, the complex interplay between different genetics and environmental factors that will be subsequently discussed triggers significant changes in the innate and adaptive immune system, driving to abnormal production of pro-inflammatory and anti-inflammatory cytokines and antibodies. This dysregulation can lead either separately or concurrently, to uncontrolled inflammation and immunosuppression, thus facilitating the development of depressive symptoms, promoting treatment resistance or enhancing the susceptibility of these patients from suffering infections, among other consequences [23]. Likewise, autoimmunity is another phenomenon closely related to mood disorders. Indeed, patients with mood disorders (especially BD) have an increased risk of suffering autoimmune maladies such as thyroid diseases and rheumatoid arthritis and in turn, patients with systemic autoimmune disorders display an increased frequency of mood disorders [25].

Compelling evidence supports that neuroinflammation is closely related to events of systemic inflammation either of acute or chronic origin [53]. One of the mechanisms by which systemic inflammation promotes neuroinflammation can be summarized as follows. First, a state of systemic inflammation characterized by elevated levels of pro-inflammatory mediators alters the permeability and function of the BBB, promoting its entry into the CNS. Then, the following mechanisms would be activated: 1) activation of neuroglia and subsequent increase of multiple pro-inflammatory mediators in the brain; 2) dysfunction of the glymphatic system and loss of polarization of aquaporin 4, which favors the accumulation of neurotoxic and pro-inflammatory substances in the brain; or 3) triggering of the febrile response in the hypothalamus (sickness behavior) and hyperactivation of the hypothalamic–pituitary–adrenal (HPA) axis [54].

To measure and study the effects and implications of immune dysfunction and systemic inflammation in patients with affective disorders, the relevance of changes in the number and profile of peripheral immune cells has been described in previous works. For example, it has been described how patients with MDD and BD present an alteration in the distribution and phenotype of circulating leukocytes of the innate and adaptive immune system compared to controls, with a bias towards pro-inflammatory profiles [55–58]. In more depth, studies appear to conclude that MDD patients have increased NK cells and decreased CD4 and CD8 T cells compared with controls [57]. Similarly, CD4 T cells appear to be polarized toward a Treg and effector T phenotype, especially with a Th17 polarization [59, 60]. Simultaneously, studies have also found that there is an increase in the number of circulating monocytes in these patients and a lower monocyte/high-density lipoprotein (HDL) cholesterol ratio [61], while other works have identified that in these cells there is an increase in the subpopulation of intermediate monocytes with a pro-inflammatory profile to the detriment of the classic ones [62]. At the cytokine level, there seems to be an increase in several cytokines such as IL-1β, IL-10, IL-6, and TNF-α, while others such as IL-8 decrease [63, 64].

Regarding BD similar changes have been observed in the CD4 T populations, with an increased polarization towards Th17, Th2, Th1, and Treg. However, strikingly it appears that these changes are even more marked than in the case of MDD [55]. Similarly, significant phenotypic changes have been described in circulating monocytes from BD patients, as well as decreased numbers of CD8 T cells compared with MDD patients and controls [65]. It is interesting to consider that the changes in the inflammatory profile can be distinctive depending on the phase of the BD. Thus, there is a much more marked dysregulation of circulating cytokines in phases of mania than in depressive phases, and the levels of some cytokines such as IL-1β can also be used to differentiate BD from MDD [66]. Likewise, recent investigations have been able to demonstrate the possible causal role of some inflammatory mediators in affective disorders, such as the IL-1 receptor antagonist and basic fibroblast growth factor with MDD [67]. This work also suggests the possible influence of suffering from BD with the dysregulation of hepatocyte growth factor and IL-9, as well as the association between stem cell growth factor beta, beta nerve growth factor, and IL-18 with MDD.

In contrast, the importance of changes in the profiles of peripheral immunoinflammatory and neuroinflammatory mediators have also been demonstrated in other less frequent affective disorders such as PMDD, in which the effect of cytokines such as IL-12, C-reactive protein (CRP), or IFN-γ is observed [68] or in SAD, in which the role of some cytokines such as IL-6, soluble IL-6 receptor, and soluble IL-2 receptor together with an increase in the polarization of macrophages towards a pro-inflammatory phenotype and CD4 T cells towards a Th1 [69, 70]. Thus, these studies reflect the existence of a clear immunoinflammatory dysfunction presents in affective disorders, manifested in the form of neuroinflammation, systemic inflammation, immunosuppression or autoimmunity. In this sense, it is interesting to highlight the bidirectional relationship between affective and immune-mediated diseases, sharing the exacerbation of the immunoinflammatory response and different genetic and environmental factors [71, 72].

**Fig. 1:**
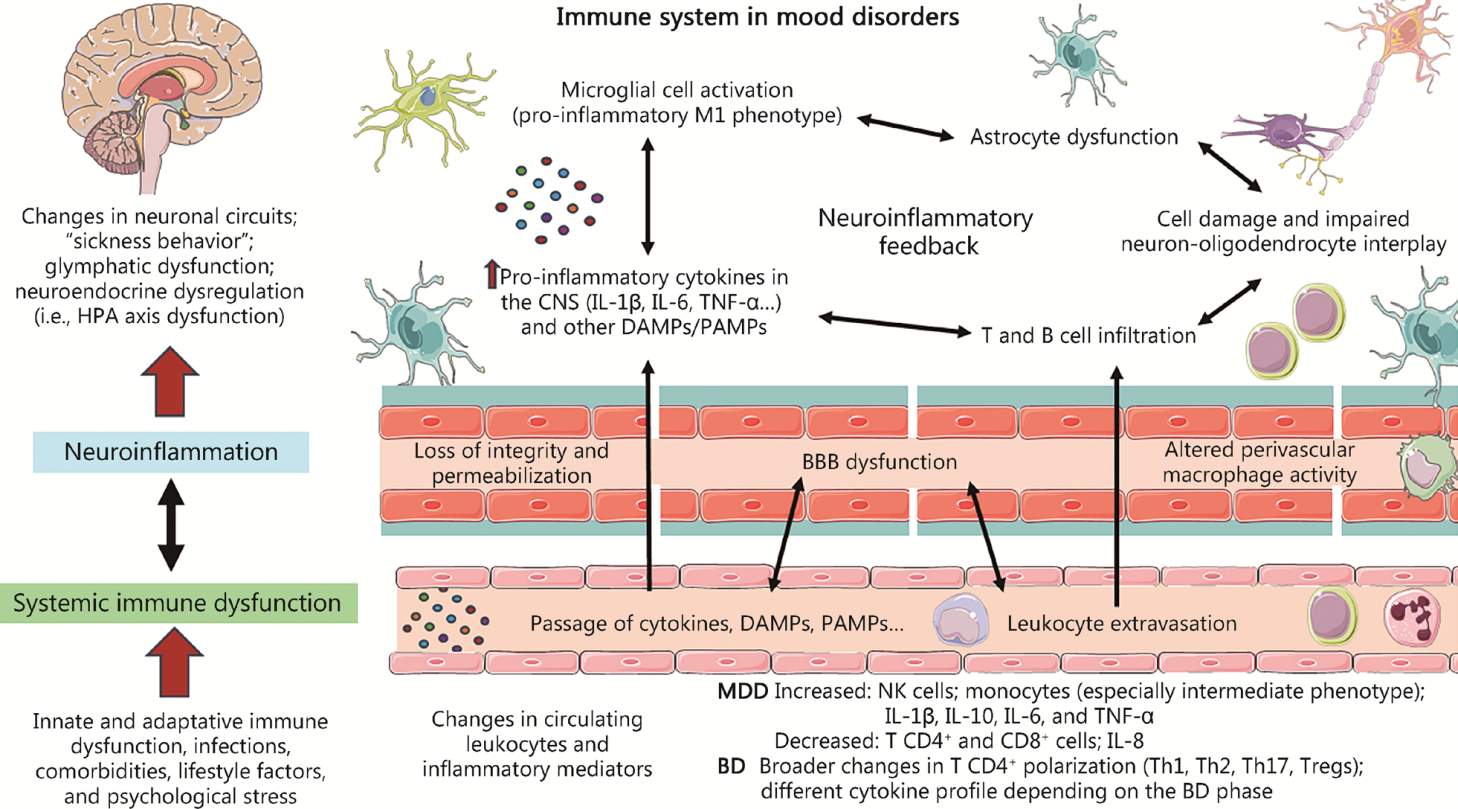
A summarized view of the role of the immune system in mood disorders. As represented both neuroinflammation and systemic immune dysfunction are the two main presentations of immune dysfunction, being tightly interconnected to each other. Peripheral inflammation, infections, and other factors enhance systemic inflammation, which can trigger neuroinflammation by promoting blood–brain barrier (BBB) dysfunction (mainly by its loss of integrity/permeabilization and altered perivascular macrophage activity), thus permitting the passage of cytokines and other factors as well as leukocyte extravasation. Once in the central nervous system (CNS), neuroinflammatory feedback occurs. This neuroinflammatory cascade is related to the pro-inflammatory environment and increased T and B cell infiltration, which are closely related to microglial cell activation, astrocyte dysfunction and alterations in neurons and oligodendrocytes, leading to a global cell damage that exacerbates this neuroinflammatory loop. Neuroinflammation is associated with changes in neuronal circuits, sickness behavior, glymphatic dysfunction, and neuroendocrine dysregulation, aiding to explain the biological basis of mood disorders. IL-1β interleukin-1β, IL-6 interleukin-6, TNF-α tumor necrosis factor-α, DAMPs damage-associated molecular patterns, PAMPs pathogen-associated molecular patterns, Tregs regulatory T cells, HPA hypothalamic–pituitary–adrenal, MDD major depressive disorder, BD bipolar disorder

Figure 1 summarizes the main mechanisms involved in neuroinflammation, systemic inflammation and immune dysfunction, as well as their interrelationship. Finally, as previously stated, the accumulated evidence has made it possible to define the relevance of a series of factors as inducers of the systemic inflammatory response, highlighting the presence of infections, inflammation of peripheral organs (with special emphasis on intestinal inflammation), the presence of comorbidities, certain unhealthy lifestyle habits, or psychological stress [54]. All these actions influence and are influenced by the immune system from the different elements of the PNIE. In the following section, the role and relationship of some of these factors with the processes of systemic inflammation and neuroinflammation observed in patients with affective disorders will be explored.

## Immune dysfunction in its context: PNIE of affective disorders

The PNIE is a crucial element in understanding the causes and consequences of immune system dysfunction in the pathogenesis of affective disorders. This section will analyze the effect of the psyche, disruption of the nervous system, endocrine and metabolic dysregulations, gut microbiota, and lifestyle factors in patients with affective disorders on the immune system.

### Disruption of the psyche in affective disorders: psychological stress and the immune system

There is a wide variety of stimuli that are interrelated with the inflammatory response in the organism. Psychological stress in two of its main aspects (stress in early life and chronic stress) is one of the most important initiators of immune responses in different psychiatric conditions, including affective disorders [73]. Early-life stress (ELS) is represented mainly by parental loss, abuse, violence, or neglect/rejection during childhood [74]. For its part, chronic stress can be caused by a wide variety of stimuli that the subject perceives as stressors that are not resolved and continue over time [75]. The different forms of psychological stress affect the body mainly through the hyperactivation of the HPA axis. This axis is composed of the paraventricular nuclei of the hypothalamus, which release corticotropin release hormone (CRH) and reaches the anterior pituitary gland, responsible for the subsequent production of adrenocorticotropic hormone (ACTH), eventually promoting the release of glucocorticoids like cortisol in the adrenal glands. Exacerbated production and release of glucocorticoids is firstly related to HPA which has a signaling effect on multiple organs and systems to redirect energy resources to meet actual or anticipated demand [76]. The underlying mechanisms related to HPA hyperactivation in mood disorders have been studied in previous literature [77]. According to the literature, described processes include 1) reduced inhibitory gamma aminobutyric (GABA) input; 2) augmented glutamatergic input from extra-hypothalamic locations; 3) reduced inhibition by the CNS, responsible for the production of arginine vasopressin; 4) stimulatory influence on the HPA axis by different factors, including neuroinflammation [76]; and/or 5) a deficient cortisol feedback effect due to the presence of glucocorticoid resistance [77]. These mechanisms lead to the disinhibition of the paraventricular nucleus of the hypothalamusand cause a chronic rise in CRH and cortisol levels, which causes mood changes through their action on the brain. In more detail, as glucocorticoids can cross the BBB, they exert long-term detrimental alterations in the brain at the structural, functional, cellular, and molecular levels [12, 78]. Glucocorticoids have direct modulatory effects on virtually all cells of the immune system. Specifically, glucocorticoids are often involved in the induction of immunosuppressive and anti-inflammatory functions through various genomic and non-genomic mechanisms, although it has also been described that they may favor both permissive and stimulatory effects on the immune system under specific conditions [79].

On the other hand, ELS has a very negative effect on the behavior and brain development of the person who suffers from it, with significant alterations in different neural networks involved in cognitive and emotional processing [80, 81]. Previous works have shown how these changes induce important alterations in the immune system, enhancing the acute and chronic inflammatory response of these subjects through the different pathways of the PNIE [82]. For instance, acute stress exposure in individuals subjected to high-stress levels in early life promotes an exacerbated sympathetic nervous system (SNS) response, which in turn is known to be a key trigger of an inflammatory response [83]. Similarly, this can also occur through exposure to physical damage or during an infectious process. In addition, ELS is associated with the dysregulation of glucocorticoid-mediated signaling in the brain and hyperactivation of the HPA axis, gut dysbiosis, and changes in certain behavior patterns such as substance abuse, sleep disturbances, or obesity [82]. All these factors are related to the development of psychopathologies such as affective disorders and the induction of a chronic systemic and neuroinflammatory inflammatory response. On the other hand, exposure to chronic psychological stress is also associated with an increase in immunoinflammatory activity, acting through the previously exposed pathways [84, 85]. Chronic or long-term stress can suppress immunity by reducing the number and function of immune cells and/or by promoting active immunosuppressive mechanisms (for example, by increasing levels of Tregs). Chronic stress can also dysregulate immune function by promoting type 2 cytokine-driven and pro-inflammatory responses [86]. Similarly, chronic stress has been described as affecting not only microglia morphology but also microglia sensitivity and reactivity [87]. Another mechanism described in the chronic stress-immune system relationship in affective disorders consists of the activation of the NOD-like receptor protein 3 (NLRP3) inflammasome in the microglia cells, inducing the secretion of IL-1β and promoting depressive behaviors [88]. Conversely, the activation of brain endothelial cells promotes the secretion of cytokines, actively participating in the inflammatory response that underlies maladaptation to chronic stress [87].

**Fig. 2:**
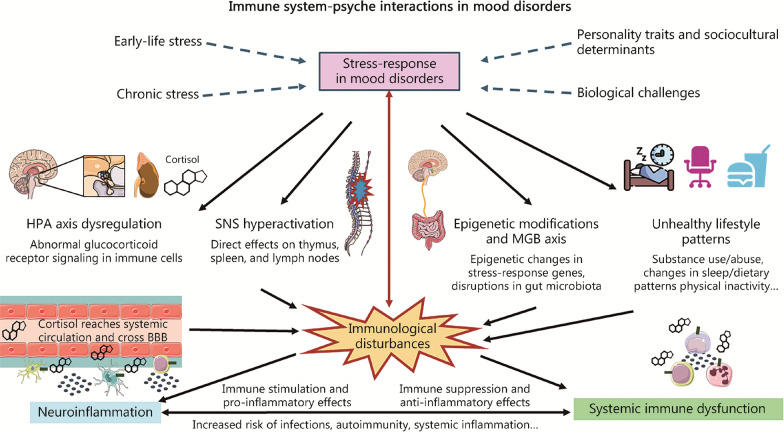
Immune system-psyche interactions in mood disorders. As shown, there is a tight relationship between the immune system and the psyche, mainly due to the hypothalamic–pituitary–adrenal (HPA) axis. Different factors such as biological challenges, sociocultural and psychological determinants are responsible for the HPA axis hyperactivation, being also early-life stress and chronic stress major triggers of this process. Then, this leads to chronic hypercortisolemia and sympathetic nervous system (SNS) hyperactivation, which among other effects leads to systemic immune dysfunction, altering immune cell status and functions and leading to increased risk of infections and systemic inflammation. As aforementioned this fact is related to neuroinflammation, but also cortisol can cross the blood–brain barrier (BBB) and lead to important alterations in the different cells located in the central nervous system by modulating glucocorticoid receptor signaling. This immune dysfunction is also considered a biological challenge, activating the hypothalamic pituitary adrenal axis, and leading to psyche manifestations. MGB microbiota gut brain

The link between stress and immune dysfunction in affective disorders are attributed to multiple pathways. First, HPA dysfunction represents the major link between psychological and sociocultural factors with biology. Psychological and sociocultural factors associated with affective disorders include different personality traits (negative self-concept, rejection sensitivity, neuroticism, rumination, negative emotionality), age, social or socioeconomic status, perceived social support, socioeconomic status, discrimination (by others or oneself because of ethnicity, beliefs or place of birth), working conditions and environment and more [89]. For a better understanding of these details, review the literature [89, 90]. Associated with HPA dysfunction, epigenetic modifications in several crucial genes have also been proposed as an important health-related effect derived from exposure to psychosocial traits [91]. For instance, epigenetic modifications in critical stress response genes like *NRC31, SLCA4, BDNF, FKBP5, SKA2, OXTR, LINGO3, POU3F1,* and *ITGB1* seem to play a critical role in MDD [92]. Epigenetic modifications can also be involved in the modulation of inflammation associated with stress in mice [93]. Another central link between psychosocial stress and immune dysfunction is throughout the aforementioned SNS. The brain projects sympathetic fibers to the primary and secondary lymphoid tissues (bone marrow/thymus and lymph nodes, respectively), releasing different substances and influencing immunological responses [94]. In this sense, the relationship between the brain with the thymus and the spleen in the context of affective disorders and psychosocial stress is notably supported by compelling evidence [95, 96]. Among other findings, an increase in thymus- and spleen-derived naïve CD4^+^ T cells can be observed in rats during the period of chronic restraint stress exposure [95], whereas splenomegaly and thymic atrophy has been observed in animal models of depression [97, 98]. In addition, psychosocial stress is associated with significant changes in the gut ecosystem, thereby influencing the immune system and contributing to the development of affective disorders through the microbiota-immune system-brain axis [99], that will be later discussed. Finally, coping with stressful experiences could also lead patients to engage in certain behaviors such as alcohol/substance use changes in sleeping patterns, diet, physical inactivity and unhealthy lifestyle habits with negative effects on the immunological system [94], collectively explaining the effects of psychosocial stress on biological mechanisms associated with affective disorder*s* (Fig. 2)*.*

### Alterations of the nervous system and inflammation in affective disorders

As previously stated, neuroinflammation is a key mechanism that helps to understand the etiopathogenesis of affective disorders. Although systemic inflammation is partly responsible for the induction of the neuroinflammatory response, there are also changes in the nervous system capable of modulating this immune system response both locally and systemically. In this subsection, the changes that occur in the nervous system and their relationship with immune dysfunction in affective disorders will be addressed.

#### Changes in the CNS and the context of neuroinflammation

As previously discussed, the CNS of patients with affective disorders exhibits significant changes at multiple levels. For example, hyperactivation of the HPA axis, dysfunction of the glymphatic system, or neuroinflammation itself are interrelated biological mechanisms that are associated with important alterations in neuronal networks located in critical brain regions such as the cingulum or the limbic system, involved in emotional processing [11].

In the same way, the changes in the levels of several neurotransmitters that occur in these subjects, especially serotonin, dopamine, and norepinephrine, have represented a key point of study in affective disorders, rising as the main target of many of the treatments currently available [100, 101]. Although it was traditionally thought that monoamine dysregulation was responsible for the pathogenesis of affective symptoms, it is now known that they are both promoters and a consequence of the various changes that occur in these patients [102]. Several studies have shown a relationship between the levels of these neurotransmitters and the immune system. The direct effect that monoamines have on mood (especially on emotions related to stress) can lead to hyperactivation of the HPA axis or dysfunction of the glymphatic system, leading to an increase in systemic inflammation and the neuroinflammatory response, which consecutively decreases monoaminergic neurotransmission [103]. Similarly, cells of the immune system express multiple components of the monoaminergic system, demonstrating the effect of monoamines not only in the CNS but also at a systemic level [104]. In this sense, one study has found an association between pro-inflammatory changes in the immune system with alterations in the metabolism of monoamines towards more cytotoxic routes in patients with BD [105]. Simultaneously, it has been possible to identify how patients who do not respond to monoamine-modulating drugs tend to present an increase in inflammatory markers compared to responders [106]. Thus, the relationship between monoaminergic changes and the inflammatory status of these patients is evident, exerting a bidirectional interaction. However, they are not the only deregulated neurotransmitters in affective disorders with an effect on the immune system.

Glutamate is another neurotransmitter that may play an important role in the pathogenesis of these conditions, being responsible for a cellular event called excitotoxicity [107]. Excitotoxicity is a mechanism of cell dysfunction and damage due to increased glutamate-mediated stimulation of neurons, altering neuronal circuitry and behavior. According to previous work, the immune dysfunction associated with affective disorders promotes the release of glutamate by glial cells, and glutamate in turn influences the function of microglia, which together with neuronal death associated with excitotoxicity promote the neuroinflammatory response in the brain [108]. Moreover, postmortem study has also found functional changes in GABAergic interneurons and a drastic reduction in the number and density of oligodendrocytes, associated with neuroinflammatory processes [109].

Moreover, the cholinergic system also plays a highly relevant role in affective disorders, having linked the increase in acetylcholine in some regions of the CNS such as the hippocampus with depressed mood [110]. The cholinergic system also has an outstanding effect in modulating inflammation. At a systemic level, the increase in cholinergic tone can act from the cholinergic anti-inflammatory pathway, a neuro-immune regulation mechanism in which the increase in acetylcholine by nerve cells (mainly from the vagus nerve) can activate the alpha 7 nicotinic acetylcholine receptor on cells of the immune system, decreasing the production of pro-inflammatory cytokines [111]. Thus, previous work has shown how patients with MDD present a decrease in some components such as butyryl-cholinesterase associated with increased cholinergic tone and its anti-inflammatory pathway, with a decrease in microglia activation [112]. However, this increased cholinergic activity was also associated with increased peripheral production of pro-inflammatory cytokines (i.e., IL-6). Thus, this receptor and this anti-inflammatory cholinergic pathway represent a very attractive therapeutic target with potential antidepressant effects, although more studies are required to allow a better understanding and therapeutic approach to this component [113].

Oxidative stress also represents a key mechanism in the nervous system-immune system interconnection. Oxidative stress consists of an imbalance between oxidizing molecules and reducing molecules (antioxidants) in favor of the former [114]. Oxidative stress is partly due to previously described neuroinflammation mechanisms and, in turn, neuroinflammation is a consequence of oxidative stress [115]. Oxidative stress and neuroinflammation also promote epigenetic reprogramming of key brain areas implicated in affective disorders, which occur through modifications to DNA, histones, or non-coding RNA such as microRNAs [116–118]. Similarly, oxidative stress is also associated with other mechanisms such as excitotoxicity, having described a wide variety of increased oxidative stress markers in these patients with decreased antioxidant systems such as glutathione, vitamin E, zinc, coenzyme Q10, or the enzyme glutathione peroxidase [119].

Finally, there are other neuromodulators altered in affective disorders such as brain-derived neurotrophic factor (BDNF) or neuropeptide Y that also have important effects on the immune system. Both components counteract the detrimental effect of immune system dysfunction in several key brain regions in these disorders, mediating multiple processes such as neurogenesis, neuroprotection, or neuroplasticity [120, 121]. However, it has been reported that the CNS and systemic levels of these markers tend to be decreased in patients with affective disorders, thus losing the immunomodulatory effects of these components [122, 123]. For this reason, the study of these components is being evaluated both as biomarkers and potential therapy in translational research, although more studies are still required in these fields [122, 124]. These changes are not the only ones highlighted at the molecular level. Similarly, it is known that a wide variety of neuropeptides such as oxytocin, substance P, or vasopressin are altered in affective disorders, although their relationship with immune system dysfunction requires further study [125].

Thus, the dysregulation of several neurotransmitters such as monoamines or glutamate, oxidative stress, and the decrease in some agents such as BDNF or neuropeptide Y, among others, represent a key point of study to understand the relationship between neurological and inflammatory changes associated with affective disorders. Likewise, it is important to highlight that the chronification of neuroinflammatory processes and neurological damage in these patients promote aging and deterioration of the CNS, helping to explain the association between affective disorders and neurodegenerative pathologies [126, 127].

#### Dysfunction of the autonomous nervous system and the association with systemic inflammation

The autonomic nervous system (ANS) regulates involuntary bodily functions like breathing, heartbeat, and digestion. It consists of two main branches: the sympathetic ANS, which triggers the fight or flight response, and the parasympathetic ANS, associated with rest functions [128]. Dysfunctions in the ANS play a crucial role in the pathogenesis of psychiatric disorders, especially affective disorders [129]. Affective disorders induce important changes in the regulation of ANS, with an increase in sympathetic vs. parasympathetic tone, frequently demonstrated by a decrease in heart rate variability [130]. The ANS is also closely linked with the CNS and the immune system. The dysfunction of the ANS and the greater activation of the sympathetic branch compared to the parasympathetic branch are mainly due to the hyperactivation of the HPA axis, which is also associated with a decrease in vagal tone, both changes being responsible for alterations in the systemic immune response [131].

Thus, this increased activity of the SNS is associated with an increase in numerous inflammatory markers and, in fact, ANS dysfunction together with exacerbated inflammation have been studied as a key etiological link between affective disorders and cardiovascular disease, which represent one of the leading causes of mortality in subjects with affective disorders [24, 132, 133]. Thus, the heart rate variability is presented as a key point of study to understand the effect of ANS dysfunction and inflammation on the risk of cardiovascular events in patients with affective disorders. Due to the potential benefits of modulating both components and the need for deeper knowledge in this field, we encourage more studies in these lines that allow appropriate translational approaches in these patients.

### Endocrine and metabolic disruptions as modulators of the immune system in affective disorders

#### Endocrine alterations

The dysregulations that occur at the metabolic and endocrine levels represent a critical point in understanding the immunoinflammatory dysfunction associated with affective disorders. The immune system-endocrine system relationship has been extensively described in the literature [134, 135]. More in detail, it has been described that there is a great variety of endocrine products (hormones) that exert pleiotropic effects in the different tissues and systems of the organism, including immune cells, where they exert a regulatory effect on polarization, proliferation, activation, and on the memory attributes of immune system cells [136].

In addition to the aforementioned glucocorticoids, there is a wide variety of peptide hormones and amino acid derivatives that exert these important actions, such as thyroid hormones and growth hormones (GHs), prolactin, dopamine, and thymopoietin, among others [136]. Alternatively, the responses of the immunoinflammatory system (for example, through the production of cytokines and other mediators) are capable of key modulating the function of the different glands and endocrine cells of the organism [137]. Simultaneously, the cells of the immune system are capable of producing many of these and other hormones, although unlike the endocrine system, these products tend to act in an autocrine manner, being able to also transport and release the hormones at local levels in different tissues after being attracted by inflammatory stimuli, thus also acting in a paracrine manner [138]. It has been described how patients with affective disorders present significant dysregulation in numerous neuroendocrine axes, including the HPA axis, hypothalamic–pituitary–thyroid (HPT), hypothalamic-pituitary-somatotropic (HPS), and hypothalamic–pituitary–gonadal (HPGn) axis [139].

##### HPT axis

In more detail, it has been described how patients with MDD tend to have increased levels of thyrotropin (TSH) together with a higher ratio of antithyroid antibodies and elevated levels of thyrotropin-releasing hormone (TRH) in the cerebrospinal fluid [140, 141]. The relationship between the HPT axis and the immune system is bidirectional. Leukocytes produce an alternative variant of TSH (TSHβ), being able to migrate from the red bone marrow to the thyroid gland and increase the levels of this hormone locally, thus modulating the HPT axis. Furthermore, the uncontrolled release of TSH can affect the different cells of the immune system, which have receptors for TSH, which can eventually promote the development of autoimmune diseases directed at these components of the HPT axis [142]. In this line, there are very interesting works that have related alterations in this axis with affective diseases such as MDD or BDs, either due to excess (hyperthyroidism) or deficiency (hypothyroidism) [143]. Hypothyroidism seems to be associated with a state of chronic depression, and more specifically due to having a depressed mood and a low lymphocyte count; while in situations of hyperthyroidism, people tend to be emotionally reactive, hedonic and impulsive, with a greater number of lymphocytes compared to hypothyroidism [14]. Importantly, the patient’s perceived social support seemed to be important in the interaction between the immune system and the HPT axis [14], demonstrating once again the integration of the immunoinflammatory response in the different elements of the PNIE.

##### HPS axis

Regarding the HPS axis, it is known that numerous immune subpopulations present GH receptors and how different organs and lymphoid structures such as the thymus, spleen, and peripheral blood produce this endocrine product. GH is produced in the pituitary in response to GH-releasing factor (GHRH), its release being modulated by somatostatin [144]. GH binds to its receptors present in different cells and tissues and controls cell proliferation mechanisms, either directly or directly from the induction of the so-called insulin-like growth factor-1 (IGF-1) [145]. IGF-1 is produced mainly in the liver, and is responsible for promoting cell growth in an endocrine, paracrine, and autocrine manner, binding mainly to its receptor (IGF-1R) or to the insulin receptor [146]. It has been suggested that both GH and IGF-1 may be potentially involved in the development of affective disorders such as MDD [147, 148]. In fact, it seems that the relationship between both hormones with affective disorders lies partially in their immunomodulatory effect. Thus, GH has been reported to have crucial immunomodulatory effects, stimulating T and B cell proliferation, immunoglobulin synthesis, myeloid progenitor cell maturation, and also modulation of cytokine responses [149]. The binding of IGF-1 to its receptor promotes the phosphorylation of the adapter protein insulin receptor substrate-1, activating the mitogen-activated protein kinases (MAPK) and phosphatidylinositol 3-kinase (PI3K) signaling pathways, thus exerting important immunomodulatory effects [150, 151]. Patients with affective disorders tend to present altered peripheral levels of IGF-1 with respect to controls, and although its implications are not well understood, it is known that this product is capable of crossing the BBB, especially in conditions of neuroinflammation [152]. Therefore, its role as a systemic immunomodulator and in the CNS of these patients deserves further investigation.

##### HPGn axis

For its part, the relevance of the HPGn axis and sex hormones in the immune system has also been described. First, it is known that immune cells produce and express gonadotropin hormone receptors, thus acting in an autocrine manner [153]. Similarly, primary lymphoid organs and cells of the peripheral immune system show marked expression of estrogen and androgen receptors, thus showing their modulatory effect. In general terms, estrogens have immunopotentiating effects, while androgens such as testosterone are associated with immunosuppressive mechanisms [154]. Previous works have observed how women tend to have a higher risk of suffering from different types of affective disorders, with higher estrogen levels being a possible explanation for this fact [155–157]. In fact, there are some subtypes of affective disorders that occur only in this group and that are mainly attributed to the action and levels of sexual hormones throughout the life of the woman. In this sense, premenstrual syndrome and its most severe manifestation previously described stand out, PMDD, which can occur in the luteal phase of the menstrual cycle due to the action of the hormone progesterone, affecting the GABAergic and serotonergic neurotransmission system [158]. Although more efforts are still required in this field, previous studies have shown the existence of an exacerbated immunoinflammatory response in this group of women compared to those who do not present these manifestations, highlighting an increase in serum levels of TNF-α, CRP, complement protein C4, and malondialdehyde, a marker of oxidative stress [159–161]. Other types of affective disorders specific to women include postpartum depression (PPD) and perimenopausal depression. In the case of PPD, it seems that changes in allopregnanolone (a metabolite of progesterone with antidepressant and anxiolytic activity) interfere with the GABAergic system and promotes the development of depressive symptoms after childbirth, while perimenopausal depression is more associated probably with changes in estradiol levels [155]. An elevation of different immunoinflammatory parameters is equally implicated in the etiopathogenesis of PPD and perimenopausal depression [162–164], demonstrating the relationship between variations in the levels of female sex hormones and the immunoinflammatory response. Moreover, it has been described how men suffering from affective disorders such as MDD also present an increase in estrogen levels accompanied by a reduction in testosterone levels [165], although exacerbated testosterone levels and a drastic reduction in estrogens can also be found in subjects with affective disorders [166]. Despite the existing evidence in this field, more studies are still required to clarify the relationship between the HPGn axis and the immune system in affective disorders.

#### Metabolic changes

By contrast, it has been described how there is a bidirectional interaction between metabolism and the immune system, responsible as a whole for the development of different pathologies such as obesity, type 2 diabetes mellitus (T2DM), or metabolic syndrome [167]. Thus, this relationship between metabolism and the immune system can contribute in the same way to the development of affective disorders. De facto, some authors have called this close relationship “affective-metabolic syndrome” [168]. Among the multiple elements that can connect metabolism, the immune system, and neuropsychiatric disorders, we will highlight the metabolic profiles of carbohydrates and lipids, including insulin and adipose tissue, as well as the state of the musculoskeletal system.

##### Metabolic alterations in glucose and lipid homeostasis

Maintaining homeostasis in glucose metabolism is essential for the modulation of immune system cells, since both an excess and a deficiency in its regulation have a very negative impact on these cells [169]. Alterations in glucose metabolism are elements commonly observed in patients with affective disorders and other mental pathologies, which predisposes them to suffer associated diseases such as T2DM [170]. In order to understand the working relationship between glucose metabolism, the immune system, and affective disorders, we must include the importance of insulin as a key element. In fact, some authors have defended the importance of insulin resistance as a common etiopathogenic mechanism between depression and T2DM [171], while other works have shown that approximately 50% of patients with BD present resistance to this hormone [172]. Insulin, in addition to being one of the hormones responsible for maintaining glucose levels in the body, has important modulatory effects on a wide variety of cells that are not directly involved in glucose homeostasis. For example, cells of the immune system possess receptors for insulin, which acts as a key regulator of multiple immunological processes, exerting both pro- and anti-inflammatory effects depending on the context [173]. In turn, the cells of the immune system will also tightly control the release of insulin, promoting insulin resistance mechanisms [174]. Thus, inflammation together with other factors such as cortisol are raised as mechanisms associated with neurodegeneration and changes in brain energy metabolism in affective disorders, demonstrating their link with insulin resistance [175].

Changes in brain and circulating lipid metabolism are an important hallmark of affective disorders. For example, lipidomic alterations have been reported in the brain of patients with affective disorders, including disruptions at the level of phospholipids, fatty acids such as omega-3, sphingolipids, cholesterol, and endocannabinoids located in the plasma membranes of neuronal cells [176–178]. On the contrary, at a systemic level, it is known that hyperlipidemia is a highly represented condition in patients with affective disorders and how these are usually accompanied by low levels of cholesterol, both HDL and low-density lipoprotein [179]. In more detail, the low levels of circulating cholesterol and in the brain of patients with affective disorders may be involved in a dysfunction of the serotonergic system and predispose to aggressive behaviors, depressive episodes and increase the risk of suicide, although other authors defend that the reduction in cholesterol levels is an epiphenomenal consequence of these disorders [180]. In any case, many of these lipids have an important immunomodulatory effect, tightly controlling different activation and differentiation processes in cells of the innate and adaptive immune system [181].

##### Adipose and skeletal muscle dysfunction

Other lines of research have also highlighted the direct relationship between the levels of visceral adipose tissue with the risk of suffering from affective disorders [182]. This association appears to be bidirectional and frequently occurs with lower levels of muscle tissue (sarcopenia) which, together with the increase in visceral fat, promotes a chronic pro-inflammatory state that promotes the development of affective disorders [183]. In fact, according to a systematic review performed by Gharipour et al. [184], chronic inflammation associated with high visceral adipose tissue seems to activate epigenetic reprogramming of several products involved in the development of MDD, such as TAP binding protein, BDNF, and Sorbin and SH3 Domain Containing 2. Thus, inflammation is defended as a key link between obesity (body mass index > 30 kg/m^2^) and affective disorders, although the metabolic dysregulation of these patients in other aspects also contributes to their development [185]. It is also interesting to explore the hormones produced by adipose tissue (adipokines) and their relationship with affective disorders. In this sense, anti-inflammatory adipokines such as adiponectin or resistin appear to be decreased in patients with MDD, while leptin tends to be increased [186]. Simultaneously, BD patients tend to have higher levels of adiponectin, leptin, and soluble TNF-α receptor 1 [187]. Other studies, however, have found how adiponectin could be used as a marker of the BD phase as there is a decrease in its levels in depressive phases, mainly associated with metabolic and inflammatory dysfunction [188, 189]. Interestingly, some authors have found that there is a decrease in adiponectin levels during the winter in patients with SAD, collaborating with the pro-inflammatory and depressive status of these patients [190]. However, there is a great heterogeneity in the studies and a greater control of confounding variables such as body mass index, the severity of the symptoms and the type of study should be considered in future research to establish a possible clinical use of these markers.

**Fig. 3:**
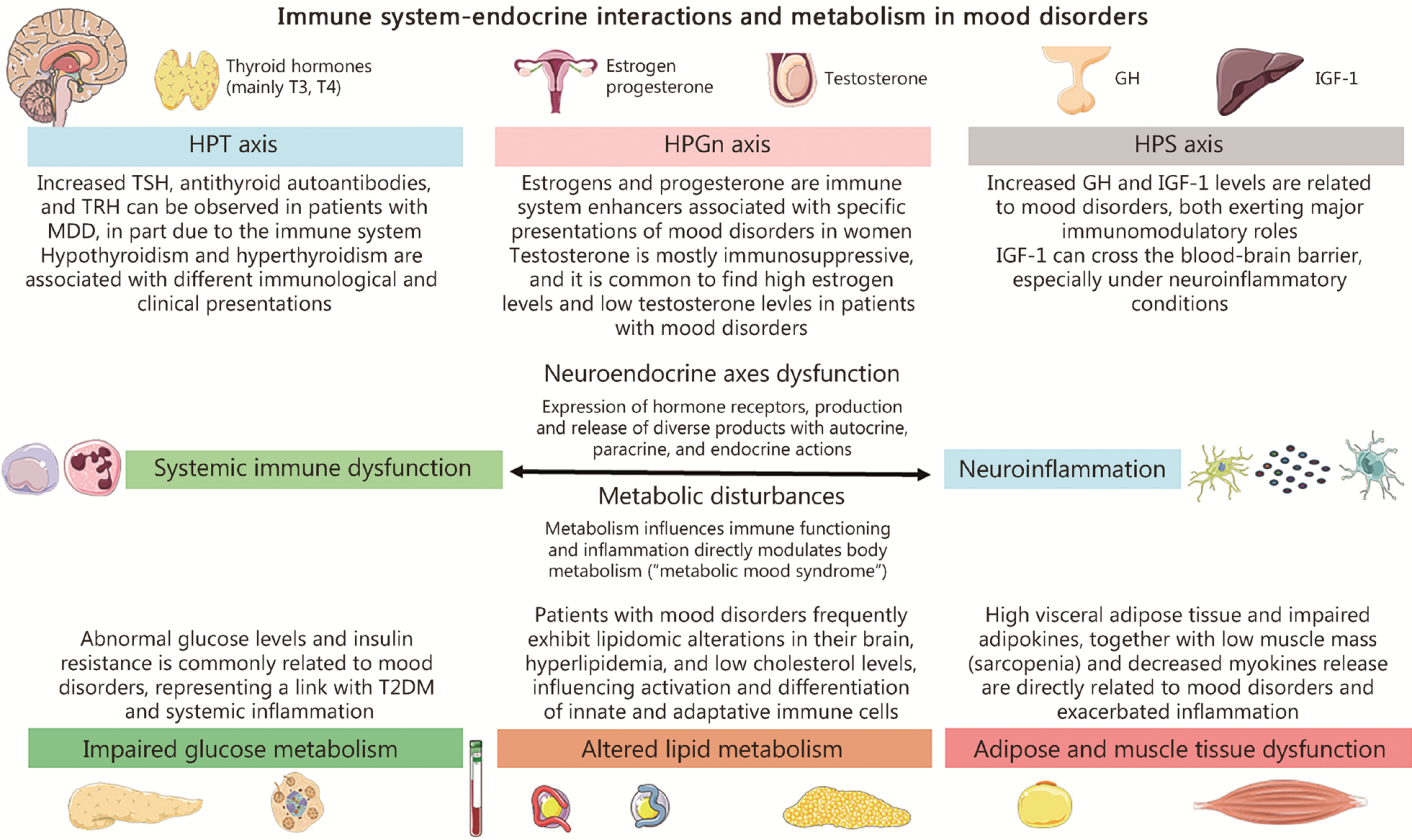
Immune system-endocrine interactions and metabolism in mood disorders. In the upper part of the picture, the main neuroendocrine axes altered in patients with mood disorders are summarized (without including the hypothalamic pituitary adrenal axis). These alterations are tightly linked to systemic inflammation and neuroinflammation in a bidirectional relationship, as these neuroendocrine products can have direct immunomodulatory effects but also the immune system can modulate and impair the neuroendocrine axes. A similar relationship is observed between the metabolic disturbances and immune dysfunction, especially when considering glucose and lipid metabolism as well as adipose and muscle tissue dysfunction. A summarized view of each section is included in the proper figure. T3 triiodothyronine, T4 thyroxine, GH growth hormone, IGF-1 insulin-like growth factor-1, TSH thyroid-stimulating hormone, TRH thyrotropin-releasing hormone, HPT hypothalamic–pituitary–thyroid, HPS hypothalamic–pituitary–somatotropic, HPGn hypothalamic–pituitary–gonadal, MDD major depressive disorder, BBB blood–brain barrier, T2DM type 2 diabetes mellitus

Additionally, sarcopenia and MDD show a direct relationship to a large extent due to the immunoinflammatory alterations they share, such as elevated levels of IL-6, CRP and TNF-α, responsible for the induction of numerous systemic changes observed in patients with sarcopenia and MDD [191]. Similarly, BD patients also have sarcopenia more frequently than the general population [192], with inflammation being a key link in the two-way relationship between affective disorders and sarcopenia. Regarding the hormones produced by skeletal muscle when training, myokines have an important anti-inflammatory role and in relieving neuroinflammation, physical exercise being a great opportunity to stimulate their production [193]. In this sense, the association between serum/brain levels of critical myokines like IL‐6 and irisin with depressive mood and impairments in quality of life has been demonstrated in previous works [194, 195]. Taken together, endocrine and metabolic changes are closely related by various mechanisms to affective disorders and immune system dysfunction, as summarized in Fig. 3.

### The intestinal microbiota, the immune system, and the microbiota-gut-brain axis in affective disorders

The intestinal microbiota is the set of microorganisms (mainly bacteria, but also viruses, fungi and even parasites such as protozoa) that inhabit the colon, especially in the final tracts [196]. Some authors consider the intestinal microbiota a unique endocrine organ due to its multiple actions throughout the organism [197]. In fact, it is estimated that for every human eukaryotic cell, there is at least one other cell of the microbiota [196], thus demonstrating the importance of these microorganisms in the functioning of the body in conditions of health and disease. The colon represents a unique and extraordinarily complex ecosystem in which the intestinal microbiota, intestinal epithelial cells, and cells of the immune system, located just below them, cohabit and interact continuously [198]. Each cell fulfills its function in the intestinal ecosystem. The microbiota for its part will be mainly responsible for participating in the regulation of the immune system, in defense against pathogens and in the metabolism of nutrients of the host [199]. In the case of this last point, the intestinal microbiota participates in the production of vitamins, amino acids, and other metabolites of great importance such as short-chain fatty acids (SCFAs), obtained from the fermentation of indigestible products such as dietary fiber [200]. For their part, the cells of the intestinal epithelium will be responsible for the absorption of nutrients and water (enterocytes), and for the modulation and communication between the body and the intestinal microbiota through the production of mucus (goblet cells); promoting the translocation of the microbiota from the intestinal lumen to the cells of the immune system located in the lamina propria (M cells); acting as microbial sensors (tufted cells); or by participating in the secretion of antimicrobial products (Tuft and Paneth cells in the small intestine) [201, 202]. Besides, enteroendocrine cells are located throughout the gastrointestinal tract, participating in secretion and motility, the regulation of food intake, postprandial glucose levels and metabolism [203]. The cells of the immune system, conversely, will receive microbial signals (either by the translocation processes mentioned above or by the production of different metabolites such as the SCFAs themselves), developing an appropriate response to the signals received tolerance or inflammation [204–206]. The cells of the immune system are grouped in the lamina propria of the intestine forming what is known as gut-associated lymphoid tissue (GALT) [207]. Under physiological conditions, this relationship between the different elements of the intestinal ecosystem is in a dynamic equilibrium, associating an imbalance with an endless number of pathologies [208]. In fact, an altered state of the intestinal microbiota characterized by changes in microbial populations or in their functioning is what is known as dysbiosis, a condition that is closely associated with intestinal inflammation and systemic inflammation, thus affecting the whole organism [209, 210].

Similarly, the intestine is especially innervated by different branches of the ANS, highlighting the enteric nervous system and the vagus nerve. The enteric nervous system, for its part, is responsible for controlling multiple intestinal functions and connects directly with fibers of the sympathetic and parasympathetic nervous system [128]. For its part, the vagus nerve is a branch of the parasympathetic nervous system that directly connects the intestine with the brainstem, which in turn connects bidirectionally with higher brain centers, including the limbic system, thus participating in the regulation emotional, sensory, and motor functions [211]. The vagus nerve-intestinal ecosystem communication is also bidirectional and mutually influential. However, interestingly, it is known that the vagus nerve is a mixed nerve composed of 90% afferent nerve fibers, that is, most of the fibers of this nerve send information from the intestine to the brain [212]. Likewise, there is innervation of parasympathetic fibers from the sacral regions, which innervate the distal third of the colon, and sympathetic fibers from the splanchnic nerves, interacting as has been said with the enteric nervous system [213]. In this way, the relationship between the intestinal ecosystem and the brain occurs directly through the vagus nerve, through the microbiota-immune system relationship, and through the action of multiple metabolites released both by the microbiota and by other cells of the intestinal mucosa, that can cross the BBB. This relationship is known as the microbiota-gut-brain axis and for this reason many authors consider the intestine as the “second brain” [214, 215].

Accumulating evidence supports the importance of dysfunction of the microbiota-gut-brain axis as a key driver of affective disorders [216–218]. Within the multifactorial picture presented by this type of psychiatric conditions, the disruption of the microbiota-intestine-brain axis can be studied from its three main aspects previously developed. In the case of gut-brain communication by the vagus nerve, the influence of changes in the intestinal ecosystem on the induction of affective disorders has been demonstrated. This occurs in two ways: 1) by affecting its afferent pathways, which sense the disruption that has occurred in the intestinal ecosystem, and 2) through interference in the efferent pathways (decreased vagal tone) closely associated with hyperactivation of the intestinal HPA axis [219]. These changes promote the disruption of the intestinal barrier, favoring dysbiosis, bacterial translocation, intestinal and systemic inflammation, and ultimately promoting neuroinflammation, again contributing to the pathophysiology of affective disorders [219]. For this reason, vagus nerve stimulation represents a therapeutic pathway that is currently being explored in patients with affective disorders resistant to treatment, although there is still a long way to go before its establishment in clinical routine [220, 221]. In the wide dialogue gut-brain, it is of note to mention the recently discovered “neuropod cells”, specialized sensory epithelial cells that synapse sensory signals from the small intestine and colon mucosa to the brain, via peripheral nerves connected to the vagus nerve and therefore the CNS [222]. In other words, it is a subset of enteroendocrine cells that form a neuroepithelial network able to transduce gut luminal signals to the brainstem [223]. Thus, the dysregulation at this level is just another piece of the puzzle of complex affective disorders.

Regarding the intestinal dysbiosis observed in patients with affective disorders, studies show how, in general, they present a decrease in microbial diversity with changes in certain microbial populations, some of them common and others exhibited differentially [224, 225]. For example, it has been shown how mental disorders tend to share a reduction in bacterial genera that produce SCFAs, besides an increase in lactic acid-producing bacteria and bacteria associated with glutamate and GABA metabolism [226]. Simultaneously, increased fecal levels of SCFAs are associated with various intestinal manifestations and depressive symptoms [227], suggesting that patients with affective disorders may have decreased production and absorption of this important metabolite. At the same time, affective disorders such as MDD and BD share specific changes in certain populations, including an increase in the levels of *Actinobacteria* and *Enterobacteriaceae*, and a decrease in *Faecalibacterium* [224], while other changes appear to be exclusive to each of these disorders, such as an increase in *Alistipes* and *Parabacteroides* and a decrease in *Prevotella* associated with MDD, and an increase in *Bifidobacterium* and *Oscillibacter* associated with BD [226]. In any case, these changes observed in the microbial composition of patients with affective disorders will also be associated with intestinal barrier dysfunction and intestinal and systemic inflammation, interacting with the immune system and cells of the intestinal ecosystem. The mechanisms by which they act can be direct, generating changes in different populations of intestinal cells or the immune system, or indirectly through the production of metabolites and different products [202, 228]. In this line, previous works have been able to demonstrate the presence of systemic markers of intestinal permeability, bacterial translocation, lipopolysaccharide (LPS; also referenced as endotoxins), and other microbial products as well as immunoinflammatory changes in patients with affective disorders. For example, patients with MDD tend to present important changes in circulating monocytes, being characterized by presenting a pro-inflammatory status, accompanied by an increased expression of IL-1β and IL-6, and an increase in serum levels of TNF-α, IL-1β, and intestinal permeability markers like LPS-binding protein (LBP) and intestinal fatty acid-binding protein [62]. Similarly, it has also been possible to demonstrate that patients with MDD show significant alterations in Treg lymphocytes, being more marked in the case of those who presented higher levels of LBP [59]. In the case of BD, study has found higher levels of circulating CD4 T lymphocytes compared to patients with MDD, including in the Th1, Th2, Th17, and Treg populations [55]. These changes are also related to the significant increase in multiple markers of intestinal permeability and bacterial translocation, as well as the previously described dysregulation of microbial metabolites that accompanies these patients [229]. For all this, it is quite common for patients with intestinal inflammatory conditions to develop affective disorders [230].

Finally, of the different altered microbial metabolites, SCFAs, tryptophan and its derivatives are the ones that will be most clearly related to the immunoinflammatory status observed in patients with affective disorders [228]. Regarding SCFAs, they are mainly attributed to anti-inflammatory functions, being able to modulate the activation and differentiation of Tregs and modulating the neuroinflammatory function of glial cells [231, 232]. However, as stated above, it is common for patients with affective disorders to present a decrease in the number of SCFA-producing bacteria and greater detection of these components in fecal samples, which would imply a decrease in the anti-inflammatory properties of SCFAs at the systemic and neuroinflammatory levels. On the contrary, in the case of tryptophan and its metabolites, the role of the intestinal microbiota is somewhat more complex. Some of the intestinal microbiota bacteria are capable of producing tryptophan, although its most important function revolves around its metabolism, giving rise to a wide variety of compounds, among which the production of different antimicrobial substances stands out, or the synthesis of tryptophan, serotonin, kynurenine (Kyn), tryptamine, and indoles [233, 234]. Although the importance of serotonin in the immune system has already been discussed previously, it should be noted that according to some studies, approximately 90% of the serotonin required for CNS functions (mood, sleep, or behavior) is produced in the immune system intestine [25], thus highlighting the role of the microbiota in this regard. Of the phyla involved in the metabolism of tryptophan, *Firmicutes, Bacteroidetes, Actinobacteria, Proteobacteria*, and *Fusobacteria* stand out, and more specifically the genera *Clostridium*, *Burkholderia*, *Streptomyces, Pseudomonas,* and *Bacillus* [235]. Although several of these metabolites have immunomodulatory action, alterations in the so-called Kyn pathway are the ones that have been most associated with alterations in the immunoinflammatory response in affective disorders, also representing up to 95% of the tryptophan degradation of the diet [236–238]. In fact, it has been shown how patients with affective disorders show a decrease in the levels of tryptophan, Kyn, and kynurenic acid (both with a neuroprotective effect), while the levels of their two main neurotoxic metabolites, 3-hydroxyKyn and quinolic acid (QA) tend to be increased in these subjects [239]. The changes that occur in the intestinal microbiota seem to play an important role in the dysregulation of these metabolites in different neuropsychiatric disorders [240]. These changes exert an important immunomodulatory effect by binding to aryl hydrocarbon receptors. The alteration of these metabolites and their binding to these receptors are potent inducers of the intestinal and systemic inflammatory response [241, 242]. Interestingly, tryptophan and Kyn can also cross the BBB, which together with the entry of pro-inflammatory cytokines and hyperactivation of the HPA axis can promote the production of some neurotoxic metabolites such as QA itself, thus favoring the neuroinflammatory cascade and altering other key processes in the brain [243]. One of the mechanisms by which these neurotoxic components act is through the synergistic effect with glutamate, promoting excitotoxicity in neurons [244]. Thus, the changes in the Kyn pathway mediated by the intestinal microbiota are a key pathogenic point to understanding affective disorders. Lastly, the role of the microbiota in the metabolism of female sex hormones and in the derived immunoinflammatory alterations is also noteworthy. This modulating role of the microbiota in hormonal levels is what is known as strobolome, representing a potential point of study in the field of specific affective disorders previously described that affect women [155]. In this sense, different investigations have shown how different populations of the microbiota specifically modulate estrogen levels from the synthesis of the enzyme β-glucuronidase [increasing the levels of free estrogen (active form)] by the synthesis of mimic compounds obtained from the degradation of polyphenols (exerting effects similar to estrogens in the body), or by influencing the enterohepatic circulation of non-ovarian estrogens [228, 245]. Nevertheless, despite the relevance of the strobolome in immunoendocrine modulation in women, several studies are still required to decipher the connection of these three components in the development and manifestations of affective disorders.

**Fig. 4:**
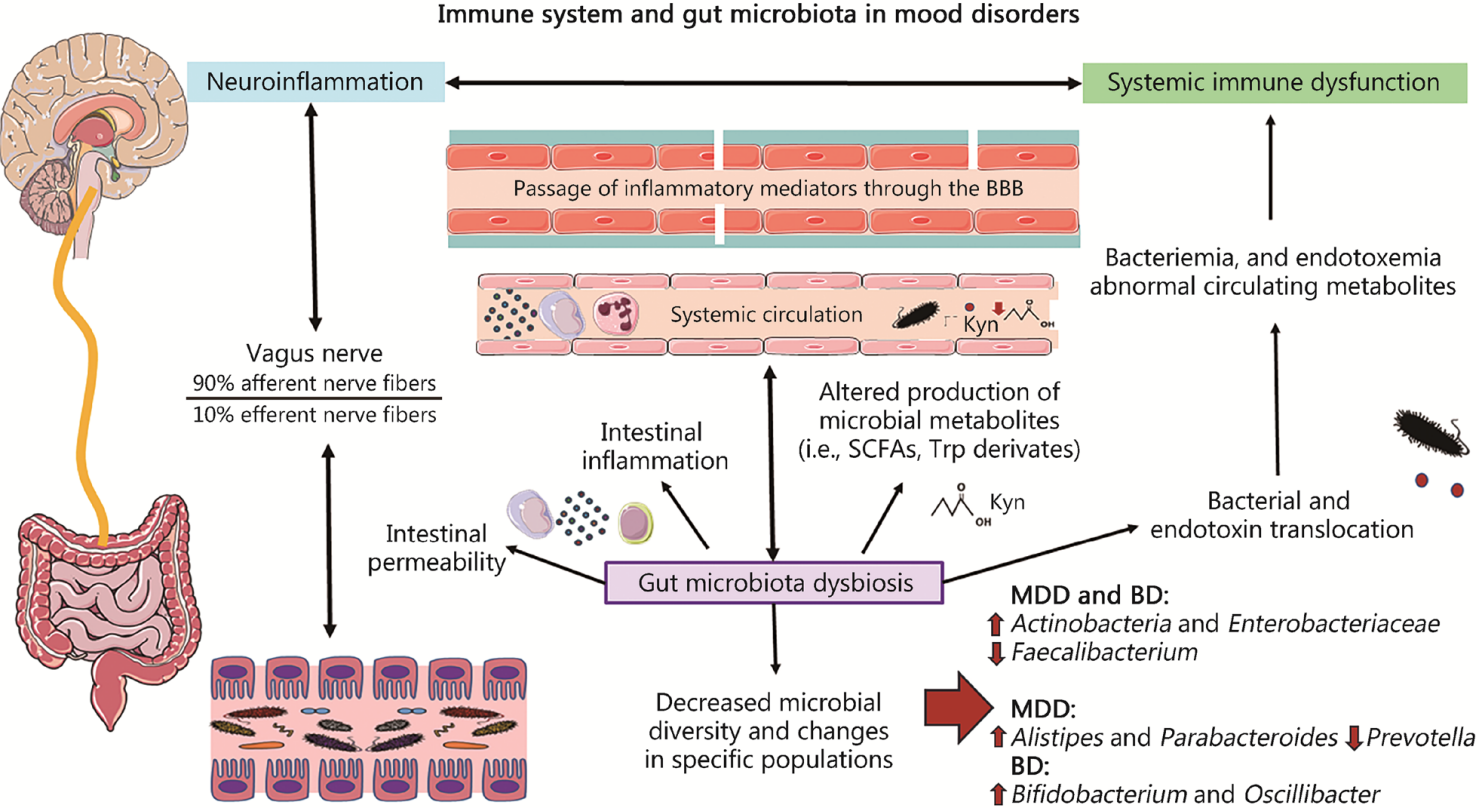
Interplay between immune system and gut microbiota in mood disorders. Gut microbiota dysbiosis is associated with decreased microbial diversity and changes in specific microbial populations. There are some microbial changes shared by major depressive disorder (MDD) and bipolar disorders (BD), although there are also particular alterations characteristic of each disorder. Gut microbiota dysbiosis is also tightly linked to intestinal permeability, intestinal inflammation, altered production of microbial metabolites, and bacterial and endotoxin translocation. Overall, these processes can affect the brain via two different mechanisms: 1) neural pathways, especially represented by the vagus nerve, a mixed nerve composed of a 90% of afferent nerve fibers, explaining the strong influence of the gut in the brain and; 2) by promoting systemic inflammation, especially through the bacteriemia and endotoxemia as well as the abnormal circulating metabolites. Many of these components can then cross the blood–brain barrier (BBB), also promoting neuroinflammation. SCFAs short-chain fatty acids, Trp tryptophan, Kyn kynurenine, MDD major depressive disorder, BD bipolar disorder

To sum up, the microbiota-gut-brain axis represents a key point of study in affective disorders, with the interaction between the microbiota and the immune system being one of the most important study frameworks for understanding the etiopathogenesis and development of these disorders (Fig. 4).

### Lifestyle as a key modulator of immune system dysfunction in affective disorders

Lastly, lifestyle is a fundamental element of study to understand the alterations of the immune system within the framework of the PNIE in affective disorders. Among these lifestyle factors are diet, physical activity, sleep, exposure to sunlight and nature, and social relationships representing fundamental translational opportunities to improve the clinical management of patients with affective disorders [246, 247]. However, it should be noted that the relationship between lifestyle and affective disorders is bidirectional. Suffering from this type of condition leads to poorer self-care and unhealthy lifestyle habits, which in turn promotes a worsening of immunoinflammatory dysfunction in these patients. Thus, in this section, we will focus on the main lifestyle habits that can promote affective disorders and their effect on the immune system, understanding the association between these components.

#### Diet

The immune system is directly modulated by the different nutrients and components of the diet and also indirectly, from the action of the diet on the intestinal microbiota [248]. In fact, diet, the immune system, and the intestinal microbiota make up a highly interesting trialogue to understand the integration of these three elements in health and disease conditions [249]. The mechanisms by which the intestinal microbiota and the immune system interact and participate in the disruption of the microbiota-gut-brain axis in affective disorders have already been described previously. However, it is essential to consider the role of diet in this context, since diet is going to be a key modulator of the microbial populations that grow in the intestinal ecosystem and of their production of different metabolites, protecting or damaging the cells of the intestinal epithelium and determining the type and intensity of the immune response that occurs in the GALT. Thus, a Western diet intake pattern, characterized by an excess of unhealthy components [refined sugars, trans fats, salt, certain additives such as emulsifiers, sweeteners (mainly sucralose and acesulfame K), and other compounds present, for example, processed meats], accompanied by the deficit of micronutrients, fiber, and quality fats and proteins, will be responsible for promoting an intestinal and systemic immunoinflammatory state accompanied by a marked dysbiosis and loss of intestinal integrity [202, 250].

Malnutrition is a problem closely associated with changes in mood [251]. It is not unreasonable to relate the Western diet pattern to countries where there is an increasing prevalence of these psychiatric disorders [252]. It is common to observe how patients with the most common affective disorders (MDD, BD) tend to show deficiencies in certain essential micronutrients such as vitamins D and B complex and minerals such as zinc and phosphorus [253–255]. Dietary patterns poor in these micronutrients seem to be crucial in the severity and duration of the disorder [256]. In the case of vitamin D, it is noteworthy that being a nutrient that is also synthesized thanks to the receipt of sunlight, patients with depression seem to be less exposed to this [257]. We must emphasize that many of these micronutrients act as cofactors of enzymes or as regulatory elements of cells of the intestinal epithelium, the immune system, the nervous system, or even microbiota bacteria. [258].

Similarly, increased intake of refined carbohydrates and other unhealthy dietary components such as trans fatty acids has also been linked to affective disorders [259, 260]. Likewise, excess of these displaces other essential macronutrients such as omega-3 polyunsaturated fatty acids (ꞷ-3 PUFA), which are required in large quantities in neuronal membranes for proper processing of nerve signals. Low levels of omega-3 in the brain, especially docosahexaenoic acid (DHA), are associated with failures in dopaminergic function [261]. Actually, an increase in the omega-6:3 ratio contributes significantly to the pro-inflammatory status that predominates in these disorders, since there will be a higher concentration of prostaglandin, leukotrienes [262], and nuclear factor kappa B (NF-κB) pathway [263] precursors. In the same way, an association has also been shown between a low intake of protein in the diet or an excess of its consumption in foods of poor nutritional quality (for example, processed meats) with affective disorders, being associated with a worse health status, deterioration of cognitive performance and an increase in pro-inflammatory parameters [264–267]. Another critical biological link between nutrition and mood disorders resides in the crucial function of vitamin D and ꞷ-3 PUFA in the synthesis of serotonin. According to the evidence [268], brain serotonin is synthesized from tryptophan by tryptophan hydroxylase 2, which is transcriptionally activated by vitamin D hormone. Eicosapentaenoic acid (EPA) seems to increase serotonin release from presynaptic neurons by reducing E2 series prostaglandins and DHA influences serotonin receptor action by increasing cell membrane fluidity in postsynaptic neurons. Therefore, insufficient levels of vitamin D, EPA, or DHA, in combination with genetic factors and at crucial developmental periods could lead to dysfunctional serotonin activation and function and may be one underlying mechanism that contributes to neuropsychiatric and mood disorders [268].

Previous work has also shown how the lack of certain nutrients at different stages of a woman’s life can be involved in the appearance of the different affective disorders described above [155]. In addition, other authors define that diet acts through other mechanisms involved in the pathogenesis of affective disorders, influencing the impact of oxidative stress, HPA axis dysfunction, epigenetic regulation of the organism, mitochondrial function, cytoprotection, or neurogenesis [258, 269]. Thus, studies show a relationship between a more Westernized intake pattern in patients with affective disorders and the immunoinflammatory dysfunction that they present, always understood within the global framework of affective disorders.

#### Sedentary lifestyle and physical activity

A sedentary lifestyle, like malnutrition, appears as a consequence of multiple factors that not only depend on the individual, but also on society itself. These factors include the increase in office jobs, the rise of different technologies that promote a sedentary lifestyle (cell phones, televisions, etc.) or the scarcity of parks or places set up to practice physical exercise [270].

Regardless of its origin, according to the World Health Organization, physical inactivity is considered the fourth risk factor for global mortality [271]. Thus, sedentary people are at greater risk of suffering from a wide spectrum of pathologies that vary from cardiovascular diseases, metabolic disorders, cancer, osteoporosis and other musculoskeletal problems, and ultimately a poorer quality of life [272]. Besides, a sedentary lifestyle is a growing problem in our society that is directly associated with the risk of suffering from different types of affective disorders [273–275]. Previous works have shown how a sedentary lifestyle is associated with an increase in the levels of various pro-inflammatory parameters regardless of other variables such as blood glucose or levels of adiposity and obesity [276, 277]. In this way, physical inactivity directly promotes various inflammation mechanisms and indirectly by promoting the accumulation of visceral fat, loss of bone and muscle mass, and the development of various pathologies that further exacerbate immune system dysfunction [278]. On the contrary, physical activity has an important anti-inflammatory effect, exerting benefits on other elements of the PNIE such as the HPA axis, the ANS, metabolism, the endocrine system, at the level of the intestinal microbiota and even in the brain itself, stimulating neurogenesis and relieving other mechanisms involved in affective disorders [279–281]. Thus, the low levels of physical activity presented by patients with affective disorders exacerbate immunoinflammatory dysfunction in patients with affective disorders, so emphasis should be placed on promoting physical activity in these patients.

#### Rest and sleep

Regarding rest, there is ample evidence of the benefits it has at the global health level, affecting, of course, the function of the immune system [282]. It is estimated that approximately 1 in 3 or even 2 patients with affective disorders have chronic sleep problems [282]. Among these sleep dysfunctions, they are observed through both subjective and objective parameters, and it is common for patients to report insomnia, hypersomnia, primary sleep disorders, or alterations in the circadian rhythm and in the chronobiology of these patients [283]. In fact, some authors consider sleep as an epiphenomenon that hinders recovery or promotes relapse in subjects with affective disorders, which in turn can promote substance abuse or suicidality [284]. The relationship between sleep problems and affective disorders is also bidirectional. This is because both conditions share many pathogenic mechanisms such as neurotransmitter imbalance in the brain, HPA axis dysfunction, and exacerbated inflammation [285].

As we indicated in the case of vitamin D in deficit due to low sun exposure, the hormone melatonin is also altered by this light–dark dysregulation due to the more time that patients spend in closed spaces than outdoors, among other factors. The organism is affected at the level of the pineal gland due to overexcitation by white lights, causing a drop in melatonin when the opposite corresponds to help regulate the circadian rhythm [286]. In this way, also the bacteria of the intestinal microbiota and every cell in our body, including the immune cells and the intestinal barrier, are sensitive to circadian dysregulation [287].

Some components of the circadian machinery mediate the activation of microglia and therefore neuroinflammation [288]. Lipid peroxidation and oxidative stress are also potentiated by overactivation of the HPA axis, which is also a consequence of sleep dysregulation. In a very detailed review, Irwin [289] reports on the relationship between sleep disturbances and inflammation as drivers of various pathologies. According to this review, among the putative mechanisms that unite these three components, different internal and external stimuli (such as those described in biopsychosocial models or the psychoneuroimmunoendocrine mechanisms themselves involved in affective disorders) promote a chronic inflammatory response that affects the architecture and continuity of sleep, which in turn promotes immunoinflammatory dysfunction together with the development of different inflammatory pathologies such as affective disorders themselves. Thus, these inflammatory mechanisms that occur both at the systemic level and in the brain lead to significant dysfunction of neuronal activity throughout the CNS, including in the regions that regulate sleep [289].

#### Other lifestyle factors

Finally, one must not forget the important role that social relationships, exposure to sunlight and nature itself have in affective disorders. Regarding the first, it is known how poor quality—much more than quantity—of social relationships, as well as scant social support are closely associated with affective disorders [290, 291]. Evolutionarily, the human being is programmed so that social relationships are a fundamental point in our lives. In particular, social relationships are essential for mental and physical health, for lifestyle habits, and even for longevity [292]. The relationship between social relationships and affective disorders are based, in part, on their effect on the immune system. Thus, it has been observed in multiple studies how individuals who perceive greater social adversities (i.e., isolation or less social support) present higher inflammatory parameters and less effective immune responses than those who are more socially satisfied [293–295]. These immunomodulatory effects of social relationships are also due to their direct effect on the brain, modulating the activity of multiple regions involved in cognition, attention, and other higher functions, as well as their ability to influence HPA axis activity [296].

Regarding the effect of sunlight and nature, the accumulated evidence shows how low exposure to both is directly associated with affective disorders [297, 298]. In the case of solar radiation, its beneficial effects at the level of the regulation of circadian rhythms, the modulation it exerts at the level of the skin and in the production of serotonin, as well as in other body systems explain its benefits in disorders affective, these effects being even more evident in some particular conditions such as SAD [299, 300]. The effect of sun exposure in increasing the production of vitamin D may help explain its benefits in the relationship between the immune system and affective disorders, although the direct immunomodulatory effects of exposure to ultraviolet A and B radiation or through the production of different neuroendocrine mediators [301].

With respect to nature, in addition to exposure to the sun, the importance of the so-called phytoncides, volatile agents produced by plants and trees as a defense mechanism that have direct and indirect immunomodulatory effects from the modulation of the HPA axis and other biological mechanisms, can be highlighted [302].

**Fig. 5:**
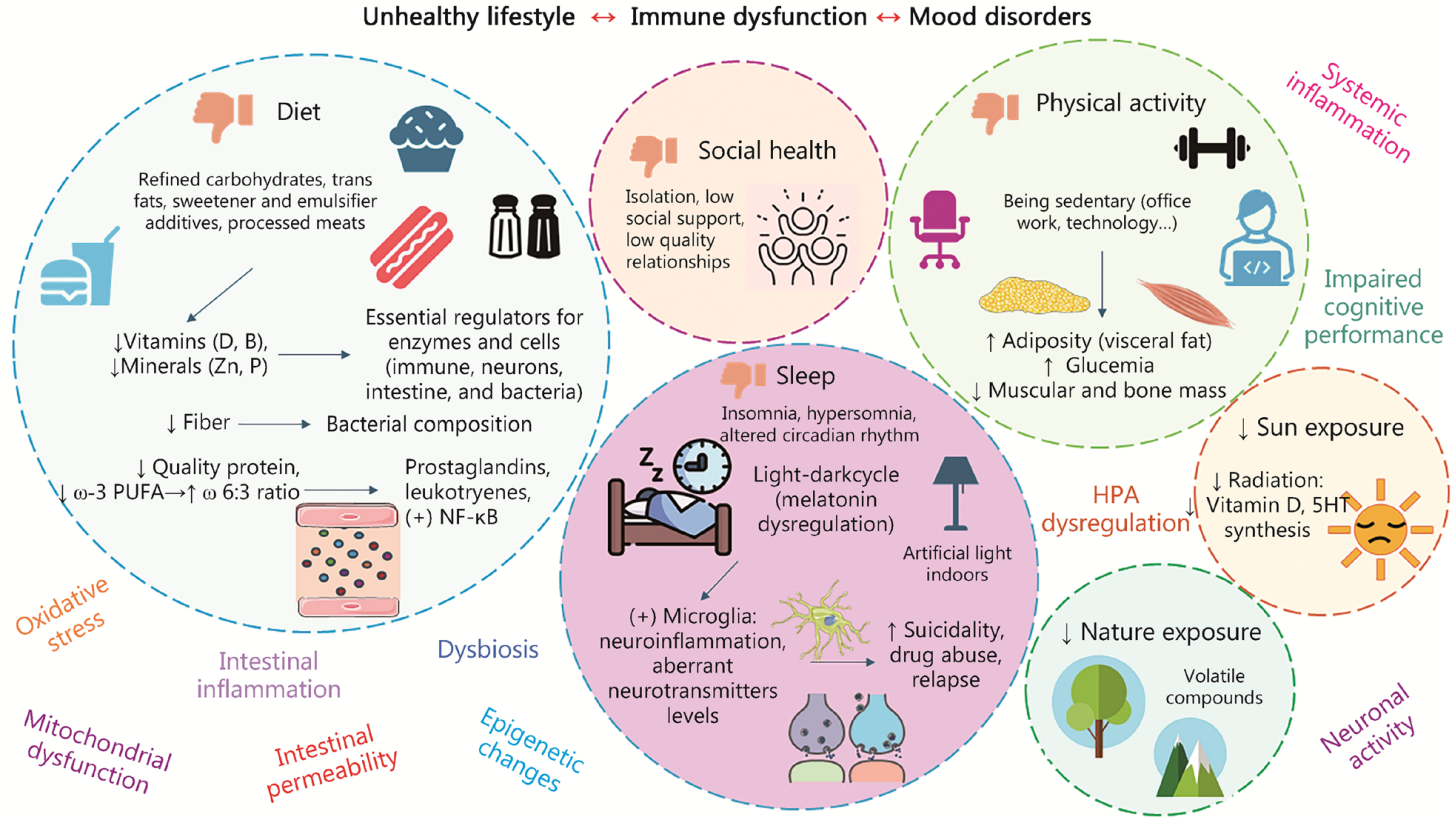
The relationship between lifestyle, immune dysfunction, and mood disorders. As summarized, diet, social health, physical activity, sleep, nature and sun exposure are major modulators of the immune system and other relevant factors associated. As explained, it is common that patients with mood disorders present unhealthy dietary patterns, with increased intake of certain components like refined carbohydrates and reduced micronutrients; sleep problems like insomnia, hypersomnia, or altered circadian rhythms; a poor quality of social relationships and isolation; reduced levels of physical activity and sedentarism and also; insufficient sun and nature exposure, missing the opportunity to positively regulate their immune system. Zn zinc, P phosphorus, ꞷ-6:3 ratio omega-6:3, PUFA polyunsaturated fatty acid ratio, HPA hypothalamic–pituitary–adrenal, NF-κB nuclear factor kappa B, 5HT serotonin

Taken together, the different lifestyle factors are a fundamental point of study that help to understand the association between immune system dysfunction and affective disorders in the context of PNIE. Figure 5 shows the main mechanisms involved in this association.

### The PNIE of affective disorders, in summary

As has been observed, immune system dysfunction represents a fundamental etiopathogenic mechanism in affective disorders, both due to neuroinflammation and systemic inflammation mechanisms, which also affect the CNS and other regions of the body. In the case of the latter, it is interesting to understand the relationship between diseases mediated by the immune system and affective disorders, sharing the exacerbation of the inflammatory response and other factors described.

However, this dysfunction of the immune system cannot and should not be understood in isolation from the functioning of the rest of the body. Thus, the impact of different events on the psyche (biopsychosocial factors) and the subsequent hyperactivation of the HPA axis is a potent inducer of the systemic inflammatory response and in the CNS, which in turn activates the HPA axis through different cytokines. On the contrary, it is also common, as previously stated, for other neuroendocrine axes such as HPT, HPS or HPGn to be deregulated for different reasons and involved in the development of affective disorders in certain patients, also interacting with the immune system.

In the CNS, other commonly observed mechanisms of damage are changes in different neurotransmitters and neuropeptides, excitotoxicity, oxidative stress, dysfunction of the glymphatic system, and different modifications at the epigenetic level in response to different mechanisms underlying affective disorders, including neuroinflammation itself. Simultaneously, these alterations are promoted and potentiated by the inflammatory response of the organism, as a whole being associated with accelerated aging of the CNS and a greater risk of neurodegenerative diseases. Likewise, the ANS dysfunction that patients with affective disorders tend to present (characterized by an increase in sympathetic vs. parasympathetic tone and a decrease in heart rate variability) is closely associated with hyperactivation of the HPA axis, being responsible for of marked inflammatory changes. These three factors together make patients with affective disorders more likely to suffer from cardiovascular diseases, explaining the comorbidity of both conditions.

As an alternative, the coexistence of affective disorders with multiple metabolic and endocrine pathologies such as diabetes or obesity is also common. This is due to the important role of metabolic changes in the regulation of glucose and lipids and their relationship with the immune system, feeding back in a bidirectional way. The intestinal microbiota is considered by different authors as a metabolic and endocrine organ, exerting a direct action on the nervous system and mood from the so-called microbiota-gut-brain axis. The intestinal microbiota interacts directly with the immune system located in the intestinal mucosa, with important effects on other cells of the intestinal ecosystem and with different nerve branches that connect with the CNS, mainly the vagus nerve. Disruption of this axis, characterized by dysbiosis, loss of intestinal integrity, and chronic and systemic inflammation, affects the CNS in multiple ways, explaining the relationship between affective disorders and inflammatory intestinal pathologies.

Finally, the lifestyle represents a fundamental link between the dysfunction of the immune system and the different elements of the PNIE. Thus, malnutrition, a sedentary lifestyle or chronic sleep disturbances have a very negative effect on the body, promoting inflammation and changes in the immune system.

**Fig. 6:**
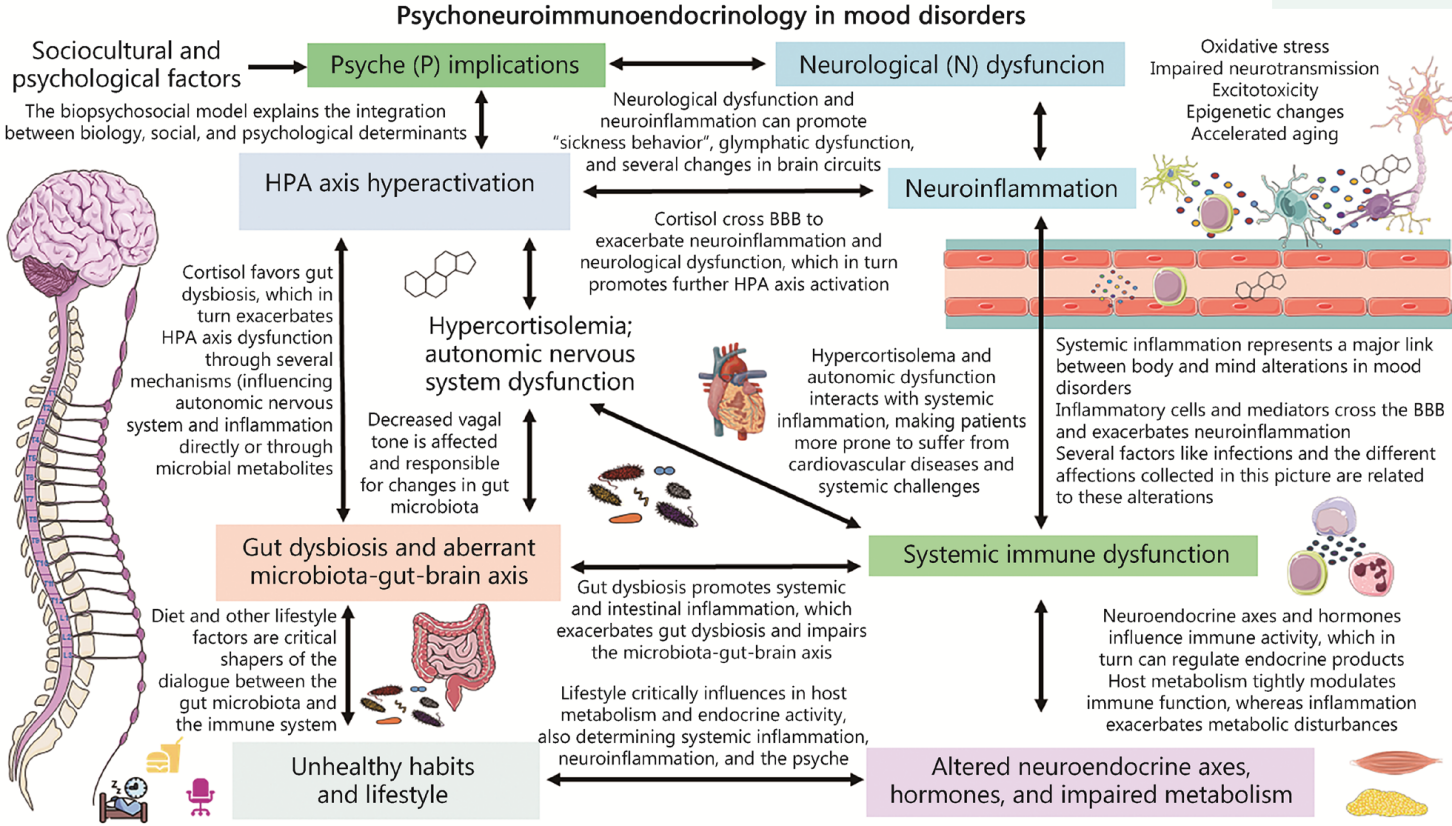
A global view on psychoneuroimmunoendocrinology in mood disorders. The role of psyche (P), neurological (N), immunological (I), endocrine (E), as well as other related systems like gut microbiota and lifestyle are summarized, aiming to integrate the different associations between all these components

Similarly, a poor quality of social relationships, low exposure to sunlight or nature are also related to inflammation and the onset and development of affective disorders. Figure 6 summarizes the different PNIE factors involved in dysregulation of the immune system in affective disorders. Despite this relationship between the immune system and affective disorders, the complexity of these systems is notable, and it will be common for some patients to show more marked or different inflammatory parameters than others. Thus, the study of the immune system as an important biomarker in precision and personalized medicine represents a very attractive point of study in current research [2]. In this sense, the relationship between different therapeutic approaches used in clinical routine and other complementary practices and interventions with potential immunomodulatory effects will be detailed below, thus trying to unravel this complex association. In this sense, the relationship between different therapeutic approaches used in clinical routine and other complementary practices and interventions with potential immunomodulatory effects will be detailed below, thus trying to unravel this complex association.

## Modulation of the immune system through clinical interventions and comprehensive treatment of affective disorders

The Greek philosopher Epictetus (55–135 A.D.), developed a paradigm of no small importance that it is necessary to internalize: “Some things are under our control, and others are not”. The biopsychosocial and psychoneuroimmunoendocrinological approaches for health and disease (in this case, affective disorders) tell us that there are alternatives that we can modulate and on which we must focus, and others that are not accessible will have to be accepted as such. All of these, considering the different biological, psychological, and social determinants, or the integration of the different systems existing in our body.

For example, we will be able to infer in an almost insignificant way in the different sociocultural determinants and in the biological mechanisms that occur in our body. However, other variables such as behavior, psychology, actions, and decisions (life habits) that one decides to take are much simpler and more effective variables to control by each individual. The role of a professional specialized in mental health is none other than to help and give tools to the person suffering from a certain affective disorder so that they find their “natural healing force”, in the words of the famous Greek physician Hippocrates.

Thus, this section will collect the main translational approaches in which interventions can be made in order to modulate the immune system of patients with affective disorders. These elements are fundamentally diet, physical activity and other lifestyle habits, psychotherapy, intestinal microbiota modulators and the use of certain pharmacological agents, also exploring the effect of antidepressants and other medications used in these patients from an immunoregulatory perspective. Although there is resistance on the part of the patients themselves and even health professionals in following this type of approach, their study and analysis are essential for the clinical and humanistic approach to these patients, as supported by scientific evidence.

### Pharmacological treatment of affective disorders and the immune system

It is vitally important to consider the close relationship that the drugs used in affective disorders have with the immune system, since this can help to understand their therapeutic success or failure in these patients [303]. The clinical management of patients with affective disorders is different depending on the diagnosis and stratification of the patient, with the DSM-5 being the guide manual par excellence used [304]. Roughly and without going into specifics, among the pharmacological options available are antidepressants, antipsychotics, anticonvulsants or some types of drugs such as lithium [3]. However, the clinical approach to the patient not only includes the pharmacological treatment selected by the psychiatrist, but rather comprehensive strategies and is combined with other approaches such as psychotherapy, lifestyle interventions and even in some more severe cases other more sophisticated techniques such as electroconvulsive therapy (ECT) [2, 3, 305, 306]. Moreover, the use of certain anti-inflammatories in the pharmacological therapy of affective disorders is also being evaluated. This section will only detail the effects of the main psychotropic drugs on the immune system and their clinical relevance, as well as interventions based on the modulation of this system.

Regarding antidepressants, there is ample scientific evidence that an important part of the benefits they exert on patients is due to their anti-inflammatory effect [307]. Among the main types of antidepressants, the selective serotonin reuptake inhibitors (SSRIs) and the selective serotonin and norepinephrine reuptake inhibitors (SNRIs) are outlined, having described several mechanisms by which they exert their immunomodulatory action. Fundamentally, it seems that these drugs exert an important benefit as antioxidants in the CNS and from the modulation of the Kyn pathway, whose relevance in the neuroinflammatory context has already been described previously [308]. Similarly, antidepressants decisively affect the intestinal microbiota, being able to act in some cases as antimicrobials and promote both beneficially and detrimentally the composition and function of the microbiota [309, 310]. Other studies have also found the effect of antidepressants in the reduction of different cytokines such as IL-4, IL-6, and IL-10, as well as IL-1β, although the variation of the latter is only observed with treatment with SSRIs [311]. Similarly, there are also studies that compared SSRIs with SNRIs, showing how venlafaxine (SNRI) shows a greater anti-inflammatory effect and different immunomodulatory actions than paroxetine (SSRI) [312]. Thus, the study of the immunomodulatory effects of the drugs used and their relationship with the immune dysfunction presented by the patient represents a potential line of study in individualized and precision medicine, although more research is required in this field that allows an approach translational in this sense.

Likewise, other lines of study have shown that high inflammatory profiles before starting antidepressant therapy are associated with a worse response to the treatment received [313]. In more detail, a meta-analysis conducted by Arteaga-Henríquez et al. [314] found that elevation of two pro-inflammatory mediators, CRP and IL-6, comprised an inflammatory form of affective disorder that was associated with worse response to serotonergic agents, with better response to other drugs such as SNRIs, modulators of dopaminergic or glutamatergic action, as well as the administration of complementary anti-inflammatory agents such as infliximab, minocycline, or EPA.

Similarly, patients with increased expression of inflammatory genes in circulating leukocytes had a poorer response to both SSRIs and SNRIs [314]. Thus, studying and understanding the relationship between the immune system and antidepressants is of great importance for the translational and clinical approach to these patients, as the scientific literature supports and demonstrates.

In the case of antipsychotics, there is also evidence of the immunomodulatory effect they exert and their relationship with therapeutic success or failure in psychiatric disorders [315]. For example, Al-Amin et al. [316] showed how treatment with antipsychotics modulated immune function in vitro by increasing the levels of the anti-inflammatory cytokines IL-4 and IL-10 and attenuating the levels of the pro-inflammatory IFN-γ. In a review prepared by McNamara and Lotrich [317], the use of atypical antipsychotics such as olanzapine, risperidone, and quetiapine exerted important immunomodulatory effects, decreasing the expression of pro-inflammatory cytokines at the systemic level and by microglia, although the benefits they exerted they were higher when combined with other immunomodulatory agents such as celecoxib, a cyclooxygenase 2 (COX-2) inhibitor, or ω-3 PUFA.

In the same way, antipsychotics also modulate the composition and function of the intestinal microbiota, exerting beneficial effects in some cases and being associated in others with some side effects derived from their use [229]. The immunomodulatory effect of lithium has also been defined in previous works, having demonstrated its efficacy both in stimulating and activating the immune response in some conditions and in relieving and reducing its hyperactivation in inflammatory conditions [318]. In the case of its use in BD, lithium seems to exert an important immunomodulatory effect on different populations of cells of the immune system, including monocytes, Th and Tregs, and having important benefits in patients who receive this therapy, preventing relapses in episodes of mania or depression [319]. However, in other subgroups of individuals, these changes do not seem to influence the course of the disease. Simultaneously, lithium also seems to suppress the expression of several inflammatory pathways such as glycogen synthase kinase 3 beta (GSK-3β), NF-κB and signal transducer and activator of transcription (STAT), resulting in a decrease in COX-2, IL-β, and TNF-α and an increase in the expression of IL-2, IL-10, and in some cases IL-4, IL-6, and other pro-inflammatory cytokines [319]. Similarly, lithium also appears to exert beneficial modulatory effects on the gut microbiota in a proportion of BD patients, promoting a favorable indirect immunomodulatory effect in these subjects [320].

Finally, although its use is not so established in the clinical practice of patients with affective disorders, the role of different immunomodulatory drugs that have shown promising results should not be ignored. Especially, non-steroidal anti-inflammatory drugs such as celecoxib itself, cytokine inhibitors, and pleiotropic agents such as minocycline or N-acetylcysteine (NAC) have been tested in several studies, mainly in combination with other therapies and/or immunomodulatory agents [18]. All these drugs act on one or multiple targets of the immunoinflammatory system, although the benefits of their use in different studies are still highly controversial. In a large-scale synthesis of clinical trial data that included 18 randomized trials and more than 10,000 patients, Wittenberg et al. [321] studied the effect of different immunomodulatory agents (targeted against IL-6, TNF-α, IL-12/23, CD20, COX-2, B lymphocyte stimulator, p38/MAPK14) compared to the use of placebo in patients with central symptoms of depression (depressed mood or anhedonia). Notably, they found that anti-IL-6 and, especially, anti-IL-12/23 antibodies showed greater effects on depressive symptoms than other classes of drugs. This supported that new immunotherapeutic agents may produce antidepressant effects in depressed patients with primary inflammatory disorders that were not fully explained by treatment-related changes in physical health.

### Non-pharmacological treatment of affective disorders and immune system

#### Psychotherapy

Psychotherapy and ECT are two therapeutic alternatives frequently used in affective disorders, being capable of modulating a wide variety of leukocyte populations and inflammatory mediators [313]. For example, in the case of psychotherapy, the efficacy of different types of interventions such as cognitive behavioral therapy (CBT) in modulating the immune system in patients with affective disorders has been described. In a meta-analysis conducted by Lopresti [322] that collected the results of 23 studies evaluating the association between CBT and inflammatory parameters in patients with depression and other pathologies, it was observed that 14 studies identified the reduction of at least one inflammatory parameter. Of these, some studies even established an association between this decrease with an improvement in depressive symptoms, also finding a relationship between inflammation prior to psychotherapy with a worse response to it. As a more representative example, other meta-analyses seem to demonstrate the reduction of IL-6 levels in patients with MDD after undergoing CBT, demonstrating a similar and synergistic effect with other pharmacological interventions [323]. However, more original and homogeneous studies are still required to clarify the immunomodulatory efficacy of CBT in these patients, as well as the establishment of protocols that can be extrapolated to clinical practice to thus achieve the desired effect [324].

Other types of approaches such as mindfulness have also been shown to have remarkable effects on the regulation of the immune system. For example, a meta-analysis [325] of 20 randomized clinical trials involving more than 1600 individuals supports the importance of this method in alleviating inflammation by decreasing pro-inflammatory proteins such as CRP or suppressing NF-κB-mediated signaling which is associated with the release of multiple pro-inflammatory cytokines. Similarly, other immunomodulatory effects to highlight would be the association with an increase in the activity and count of CD4 T lymphocytes and the stimulation of the enzyme telomerase, thus reducing the aging of the organism and the immune system [325]. In the case of affective disorders, mindfulness has been associated with a decrease in salivary levels of IL-6 and TNF-α [326]. The context in which mindfulness exerts its immunomodulatory effect is also due to its inhibitory effect on the HPA axis and the improvement of the intestinal ecosystem [327], although the precise effect of this intervention on the microbiota and the immune system must be studied in greater depth.

#### Lifestyle interventions

##### Nutritional intervention

As we have previously discussed, lifestyle remarkably shapes the physiological state of the individual. Diet, in particular, is considered by an increasing number of scientists in the biosanitary field, the factor with the greatest modulating power in the intestinal microbiota, the immune system and the link that exists between the two [202].

Nutritional therapies represent important support tools in the clinical management of patients with affective disorders. According to the meta-analysis by Firth et al. [328], which included 45,826 people, nutritional interventions can be quite useful for the prevention and relief of symptoms of depression. However, the majority of respondents in the included studies did not have diagnoses of clinical depression. Recent findings from a comprehensive meta-analysis by Xu et al. [329] indicate a link between different dietary strategies, food groups, and nutrients in the prevention and treatment of depression. Despite the high heterogeneity, lack of reproducibility, and little extrapolation that can be made from a large part of nutritional intervention studies, they do not fail to obtain favorable results that have been contrasted through animal models that explain the underlying biological mechanisms [330].

We have seen that patients with affective psychiatric disorders are characterized by significant immune system dysfunction, which in turn is associated with the different elements of the PNIE. The way to approach a nutritional strategy will be firstly limiting the excess of unhealthy products and secondly, supplying nutritional deficiencies [258]. It is, for all these reasons, important to conceive clinical therapy from an increasingly integrating point of view and consider the figure of the nutritionist as an adjuvant to fill these deficits. In order to satisfy all the nutritional requirements that favor good overall physiological functioning, it is important to promote a highly varied diet rich in fiber, vitamins, minerals, and different sources of ꞷ-3 PUFA, which can be achieved through the implementation of different strategies [202, 331]. In this sense, we will highlight the relevance of certain nutrients and components of the diet, foods and nutritional contexts that have demonstrated their benefit at an immunomodulatory level in affective disorders.

Around 1989 Dr. Stephen DeFelice introduced a new term that unified nutrition and pharmacy. That is why many nutrient and non-nutrient elements that we find in foods and food supplements are referred to as nutraceuticals, and they are those that help in the prevention and treatment of different pathologies [332]. In the field of affective disorders, a long list of nutraceuticals has been described that have shown an important antidepressant role, acting pleiotropically and favorably modulating immune system dysfunction, both relieving the neuroinflammatory response and systemic alterations [333]. The advantages that nutraceuticals present over other types of approaches is that they are easily obtainable, most of them from food. In the case of supplements, their cost is in many cases lower than pharmacological ones, and they are safe and highly tolerable, which is why they are widely accepted.

However, the physician must accompany the patient in his treatment and be aware of what he is ingesting, since a bad nutritional design may not be an adjuvant in this case, but an inconvenience for his pharmacological treatment. Once again, we highlight here the importance of a professional figure accompanying the patient in nutritional management as a complementary therapy to the treatment he receives. According to different systematic reviews and meta-analyses, the nutraceuticals that have demonstrated the most evidence as an adjuvant in MDD are: ꞷ-3 PUFA, vitamin D, S-adenosyl methionine, and methylfolate, while for BD, ꞷ-3 PUFA, NAC, and coenzyme Q10 show favorable results in depressive phases, although in manic phases there are few studies that support the efficacy of their use [334, 335]. In general, there is greater acceptance of the use of these nutraceuticals in patients with MDD, where the use of zinc, saffron and curcumin as adjuvants, or St. John’s wort as monotherapy has also been recommended [336]. Even so, the authors warn that there is a wide heterogeneity in the studies and that more research is required, especially in BD; although they defend that the relief and reduction of the inflammatory response by these agents may exert potential benefits in these patients [336].

Likewise, we must highlight the belonging of curcumin to a group of non-nutrient components or bioactive compounds derived from the secondary metabolism of plants called polyphenols, with anti-inflammatory and antioxidant properties. Some other examples are hydroxytyrosol from extra virgin olive oil and olives, resveratrol from grapes, blackberries, blueberries, and peanuts, phenolic compounds from coffee or tea; and many spices such as turmeric, ginger, cinnamon, thyme, oregano, etc. Numerous studies on polyphenols have shown different mechanisms by which they can inhibit the NF-κB inflammatory pathway and its products [337]. Many of them act on key enzymes in metabolic stress (AMP-activated protein kinase, sirtuins, mechanistic target of rapamycin, etc.) [338, 339]. Currently, there is a high interest in the manufacture of formulas rich in these compounds for their application to different inflammatory diseases (arthritis, asthma, atherosclerosis, diabetes…) [340, 341], and its consideration should not be less in psychiatric disorders. In fact, its active role in the modulation of the intestinal microbiota has aroused greater interest in its role in mental health. The underlying mechanisms studied also include the modulation of the Kyn pathway, these bioactive compounds promoting an increase in tryptophan in the Kyn:tryptophan ratio, for the microbial production of serotonin [342]. Certainly, we could list an infinite number of natural compounds that deserve attention and that exert a regulatory role on the microbiota, the immune system, or oxidative stress. In this review, we want to highlight the importance of addressing integrative therapies that can get the most benefit from different resources that may be available to the patient and that can give physicians and researchers a broader vision.

Food, unlike supplements, usually includes many nutrients that interact with each other, being able to exert different effects than when they act in isolation and enhance their benefits [343]. This term is designated as the food matrix and demonstrates the relevance of considering nutraceuticals not only as nutrients in isolation but also as part of a system in order to maximize their benefits [344]. For example, targeting the most frequent deficits, several comparative studies, meta-analyses, and review papers have shown, in the first place, that in those countries where fish consumption is higher, there are lower epidemiological rates of MDD and BD [345–347]. Vitamin D, whose receptors are expressed and associated with tight junction proteins (such as claudin-5) between enterocytes [348], can also be found in oily fish (D2 isoform), but above all through synthesis in the skin from sunlight (D3 isoform) [349]. Therefore, fish as a source of ꞷ-3 PUFA is fundamental and for this reason, the dietary recommendation to regularly eat oily fish can bring great benefits in the clinical management of affective disorders. Along the same lines, other foods of animal origin should also be considered (mainly other seafood, eggs, dairy products, and unprocessed white meats), but above all the predominance of foods of plant origin in the diet, including here a wide spectrum of fruits, vegetables, berries, nuts, seeds, and legumes, always included in a dietary context appropriate to the needs of the patient and that can generate adherence [350].

Finally, regarding the recommended dietary patterns, the Mediterranean diet is one of the most widely studied nutritional interventions in the existing literature [351]. This type of diet is based on the combination of highly complex carbohydrates rich in fiber (present in cereals, legumes, vegetables, and fruits), polyunsaturated fatty acids with antiatherogenic and anti-inflammatory properties (present in olive oil and nuts) and bioactive compounds with antioxidant properties (such as flavonoids, phytosterols, terpenes, and polyphenols) [352]. Thus, this eating pattern presents a low dietary inflammatory index, also epigenetically modulating different leukocyte populations and reducing Th cell imbalance (decreasing polarization towards Th1 and Th17), oxidative stress, cell adhesion, activity of B lymphocytes and complement proteins and improving the function of autophagy [351, 353]. Another type of dietary pattern is the so-called “anti-inflammatory diet”, which seeks to reduce silent inflammation and restore hormonal and genetic balance, generally in a 40:30:30 combination combined with caloric restriction, low omega-6 intake along with high intakes of the main ꞷ-3 PUFAs, EPA, and DHA (2–3 g/d) and the inclusion in the diet rich in colorful non-starchy vegetables contributing with adequate amounts of polyphenols to alleviate the inflammatory dysfunction [354]. Both the anti-inflammatory diet and the Mediterranean diet have demonstrated their efficacy as a potential support tool in the clinical management of affective disorders [355–358], while other strategies such as low-carbohydrate, ketogenic, or plant-based diets have also shown some benefits in alleviating mood symptoms and inflammatory response [350]. However, the current evidence calls for the need for more studies to support different dietary strategies, especially in the field of intervention in these patients. In any case, not only the dietary pattern, but also the adherence that patients have to this type of diet will be key to benefit from its implementation [356, 359]. Once again, the need to personalize the type of dietary guidelines for each patient based on their tastes and preferences is supported here, the integration of a multidisciplinary team being essential to follow and help the patient along this path.

##### Gut microbiota modulators

Regarding microbial modulation, the main components suggested are dietary fiber, probiotics, and prebiotics. Dietary fiber (found in whole grain cereals, fruits and vegetables, legumes…) consists of those complex carbohydrates that are not assimilated by the colonocytes when they reach the large intestine, but that can be fermented by the bacteria that reside there. Derivatives are SCFAs that have pleiotropic effects on multiple cells of the host, including those of the immune system, as mentioned above. This fiber exerts a prebiotic effect on beneficial bacteria for the body, that is, it stimulates their growth [360, 361]. Other prebiotics of interest are oligosaccharides such as inulin, oligofructose, lactulose, fructooligosaccharides, galactooligosaccharides, etc., which can also be found in fruits and vegetables, or in supplements fortified with said substances. The use of this type of nutraceutical through supplements is usually prescribed by a nutritionist when there is intestinal dysbiosis to accompany the recommendations for probiotics, since both elements will create synergy.

Probiotic bacteria are those that, when administered in adequate amounts, can partially colonize the intestine, producing a benefit on its health, such as *Lactobacilli* and *Bifidobacteria*. Currently, the term psychobiotic is used more in the field of psychiatry, which refers to live microorganisms that in adequate amounts can produce beneficial effects in patients with psychiatric illnesses [362]. These psychobiotics have been extensively studied in clinical trials of patients with psychiatric disorders and in animal models of these diseases, showing their effects at the level of the enteric nervous system and the immune system, modulating behavior, emotions and cognitive activity, with anxiolytic results and antidepressants [363]. Specifically, multispecies formulations of bacterial strains are often preferred in studies of mild, moderate depression, and chronic MDD. In addition, multispecies probiotics have shown anti-inflammatory effects after ingestion for at least 4 weeks, such as the decrease in IL-6 that was demonstrated in the PROVIT trial applied to depression [364]. Alternatively, comparative clinical trials have shown that groups where the combination of antidepressants (for example sertraline) is administered together with probiotics presented more favorable results than groups where only the drug was administered after a treatment of 8 weeks for both groups [365].

It is interesting to consider these nutraceuticals to assess prevention in individuals at risk, such as postpartum or perimenopausal women [155], or simply individuals who begin receiving psychotherapy for a stressful event. In a study carried out on non-depressed individuals who were administered a formula with various bacterial strains for 4 weeks [*Bifidobacterium bifidum* W23*, Bifidobacterium lactis* W52*, Lactobacillus acidophilus* W37*, Lactobacillus brevis* W63*, Lactobacillus casei* W56*, Lactobacillus salivarius* W24*, and Lactococcus lactis* (W19 and W58)], they observed that compared to the placebo group, the treated subjects had a lower rate of negative thoughts, mental rumination, and lower cognitive reactivity of discouragement [366]. Also in healthy individuals, formulas such as Ecologic®Barrier have shown through imaging studies that, in a stressful situation, probiotics could help working memory, favoring cognitive control and being associated with neuronal changes related to the intervention in the frontal cortex [367].

Nevertheless, the problem of existing intestinal permeability that occurs in these patients should not be forgotten to address. For this, in addition to promoting microbial health through probiotics and prebiotics, it is also convenient to strengthen the intestinal barrier. In this case, one of the key nutraceuticals is vitamin A (present in foods like liver and red, orange or yellow fruits and vegetables). In the intestinal mucosa, APCs recognize it and transform it into retinoic acid, which promotes IgA synthesis by B lymphocytes and T cell differentiation and directs its localization to the mucosa [368]. Likewise, this vitamin allows to regulate the concentration of Treg, Th and innate immune cells [369], in addition, the microbial composition also varies with the availability of retinoic acid, since IgA will favor the growth of beneficial bacteria and limit the growth of other opportunistic pathogens.

Finally, fecal microbiota transplantation (FMT) is another promising translational approach targeting the gut microbiota that involves the transfer of feces from a healthy donor to the colon of a patient with established pathology with the goal of restoring the normal microbiota and cure or at least improve the disease [370]. Although currently this type of therapy at the clinical level is considered for recurrent *Clostridium difficile* infections, there are several open lines of research in intestinal and extraintestinal pathologies, including affective disorders [371]. The current scientific literature includes cases of patients with MDD and BD who have benefited from receiving this type of therapy, even remitting their depressive and manic symptoms [372, 373]. Overall, FMT could be considered a safe therapeutic method with few adverse effects, although there are two main limitations to address before recommending it for patients with affective disorders. The first is related to the fact that there is still limited knowledge about the long-term results of FMT and greater efforts are needed to establish personalized protocols adapted to each patient and/or condition they suffer from [374]. The second issue is related to the donor. Accumulating evidence supports that FMT has the potential to not only ameliorate symptoms of affective disorders when healthy gut microbiota is transferred, but also to induce or aggravate symptoms if the donor shows altered gut microbiota [375]. It is essential to carry out a thorough examination of the donor with a questionnaire, interview, blood test, and stool analysis before allowing the transplant in order to reduce the risks [374].

Thus, microbiota modulators represent a promising adjuvant therapeutic approach for individuals with affective disorders, exerting a beneficial immunomodulatory effect for these patients. However, more work is required to evaluate its efficacy and application in different contexts and subjects, as well as the identification of possible markers that can be used in its follow-up or as therapy.

##### Physical activity programming and other factors: sleep, social health, and mind

Furthermore, physical activity helps in the correct modulation of other factors such as sleep hygiene and in the wake-sleep and fasting-eating cycles. Meta-analyses have shown that depressive symptoms can be alleviated by physical exercise compared to non-intervention groups [376, 377]. These studies have been tested in different age groups, from adolescents to the elderly. It not only helps physical health, but also psychologically and can synergize with other treatments, including antidepressants [376]. In older adults diagnosed with MDD along with cognitive impairment, better control of cognitive impairment has been observed through exercise [378]. Although there have been clinical trials applied to MDD and there is less data on the symptoms of BD in the different phases accompanied by physical exercise, it seems that these patients also improve their quality of life [379]. In addition, physical activity has the advantage that it promotes social health, derived from contact with other people depending on the type of activity [380]. Also this can strengthen that a greater adherence to the training is established and until it settles as a habit. Therefore, perhaps people with these types of disorders can benefit more from activities with other people, be they partners or coaches. It is worth mentioning that food choices or desires after playing sports are usually seen as healthier options in those individuals with affective disorders who have followed a training program [381]. In addition to the many benefits of sport already mentioned, it will also help reduce inflammation through caloric expenditure.

In relation to the body-mind connection, alternatives such as yoga and Pilates can also be considered. Perhaps due to its relaxation techniques, it can be considered one of the first options to introduce the practice of exercise in patients with high levels of depression, as some authors affirm [382]. Some trials confirm that due to this greater connection of the body-mind, the participants of studies on yoga and Pilates have greater satisfaction with their subjective state of health and this is the motor of the cycle of positive reinforcement [383, 384]. The same principle applies to studies in patients who have a diagnosis of affective disorder, especially MDD [385]. However, other studies in BD patients have found that not all activities can be applied to every patient; in the case of yoga, they seemed to encounter difficulties or fear of injuring themselves or suffering pain [386] and therefore, to promote their well-being, they should be supervised and find the activity that best allows them to develop adherence. Other studies on bipolar patients found greater benefits in yoga due to the inclusion of mindfulness techniques and deep muscle exercises that can be adjusted [387]. The scientific literature supports the immunomodulatory effect of yoga based on its regulatory effects on circulating cortisol and classic inflammatory markers such as CRP, IL-1β, IL-6, TNF-α, and IFN-γ, as well as other components such as telomerase, endorphins, IgA, and BDNF [388].

These activities also seem to show greater satisfaction with the perception of their own body image in adolescents with eating disorders, which can also be associated with psychiatric disorders such as the ones we are treating [389]. Large-scale study (*n* = 1258) has found a positive association between improvement in depressive symptoms with physical activity in young people between 18 and 23 years of age. In addition, favoring greater satisfaction with one’s own body image favored adherence to sport followed by better sleep quality [390]. Intervention study that address physical activity, would reinforce body esteem and prevent depressive symptoms [391]. Ultimately, according to the scientific literature, the two best ways to regulate sleep are physical activity and diet, including fasting-eating cycles and their food composition [392]. However, other types of interventions such as CBT may also be of great relevance for improving sleep in these subjects and its relationship with inflammatory dysfunction. In a study conducted by Dolsen et al. [393] in patients with type 1 BD, an association was found between higher levels of IL-6 with shorter sleep time. However, after CBT, a significant improvement in sleep was reported along with a decrease in the levels of IL-6 and other inflammatory mediators.

On the contrary, atrial transcutaneous vagus nerve stimulation has been extensively studied in sleep disorders. The results show that hyperactivation of the HPA axis significantly decreases, palliating the increased sensitivity to light during sleep. In addition, it regulates emotional circuits in insomnia, inhibiting brain areas associated with sensitivity and regulation of emotions, such as the amygdala and projections on the visual cortex [394]. Thus, all of these lifestyle-based interventions are associated with significant improvements in immune function in people with affective disorders. According to the latest data collected from World Federation of Societies for Biological Psychiatry (WFSBP) and Australasian Society of Lifestyle Medicine (ASLM) taskforce [395], lifestyle-based interventions for mood disorders represent a pivotal approach that might be implemented in daily clinical routine. The highest ratings to improve MDD were the use of physical activity and exercise, relaxation techniques, work-directed interventions, sleep, and mindfulness-based therapies (Grade 2). Interventions related to diet and green space were recommended, but with a lower strength of evidence (Grade 3). Recommendations regarding smoking cessation and loneliness and social support were based on expert opinion. However, broader research is required to find the most adequate approach to implement these different measures and maximize the benefits for these patients.

#### ECT

Finally, ECT is a type of therapy used in situations that require urgent clinical intervention or in patients with affective disorders resistant to treatment [396, 397] also presenting remarkable immunomodulatory and psychoneuroendocrine effects. Accordingly, it has been shown that ECT modulates the activity and levels of multiple neurotransmitters (like serotonin, dopamine, and norepinephrine) in the CNS, also promoting neurogenesis, and reducing hyperconnectivity and neuroinflammation [398]. Additionally, ECT has been shown to normalize the activity of the HPA and HPT axes. It seems that the changes related to ECT are dependent on the acute or prolonged exposure to this therapy. For instance, acute exposure to ECT seems to be associated with immunological changes and increased levels of cortisol and ACTH while repeated ECT does not have an additive effect on the immune and neuroendocrine functions [399]. From an immunological perspective, ECT seems to reduce the activity of microglial cells and astrocytes while modulating innate immune cells like macrophages, decreasing their pro-inflammatory properties and ameliorating exacerbated immune responses [398]. The study of the immunomodulatory role of ECT has shown some promising results in the available literature. For instance, the relationship between higher levels of IL-6 before treatment with an improvement in depressive symptoms has been reported [400]. Other inflammatory markers have also shown their usefulness in the relationship between ECT and the improvement of depressive symptoms, but only in women. In this sense, higher levels of CRP and lower levels of IL-8 before treatment are associated with a better response to this type of therapy, according to previous studies [400, 401]. Another recent study demonstrated the usefulness of longitudinal monitoring of IL-8 levels with the response to ECT regardless of gender [402], showing the relevance of this cytokine as a predictive biomarker in these patients. However, studies have not found a clear immunomodulatory effect of ECT in BD patients. For example, Şahin et al. [403] failed to find any difference in the neutrophil/lymphocyte ratio before or after these individuals underwent ECT, whereas the combined use of this therapy with some pharmacological agents such as celecoxib appears to promote a greater decrease in TNF-α than patients receiving ECT alone, although no effect was seen on other inflammatory cytokines [404].

Taken together, there is a clear relationship between the immune system and the existing therapies received by patients with affective disorders. Indeed, the study of different biological markers can potentially aid in the clinical management of mood disorders, as shown in Table 1. In general, a decrease in inflammatory parameters is associated with a better response to the treatment received, while basal levels of different immunoinflammatory markers can also be used to predict the response that a patient will have to the proposed therapy. For example, as aforementioned, stress and cortisol activation of the glucocorticoid receptor (GR) are significant drivers of mood disorder pathobiology and severity exacerbations. Recent work indicates that the suppression of systemic factors that suppress the GR nuclear translocation, including melatonin, gut microbiome-derived butyrate and BCL2-associated athanogene (BAG)-1, may determine not only the consequences of stress-driven HPA axis activation but also how the morning cortisol awakening response prepares the body for the coming day [405]. As melatonin, BAG-1 and butyrate also prevent the GR from being transported to the nucleus, where it activates thousands of genes expressing the glucocorticoid response element as well as interacting with, and modulating, other nuclear transcription factors [406], variation in these factors will modulate the consequences of stress and cortisol awakening response across body cells. Notably, it has been long appreciated that the classical BD treatments, lithium and valproate, both increase BAG-1 thereby inhibiting GR nuclear translocation [407] and possibly driving GR mitochondria translocation [408]. This provides one means to integrate PNIE data across mood disorders and gives a perspective to shape future research. However, more studies are needed to analyze the mechanisms by which treatments modulate the immune system and the effects of the immune system as determinants of therapeutic success or failure of a specific therapy.

**Table 1:** Translational applications of critical biological markers based on PNIE interventions

Biological marker	Translational applications	References
IL-1β	Predictive marker: variation observed primarily with treatment using SSRIs; reduction associated with antidepressant therapy	[311]
IL-4	Prognostic marker: increased levels associated with the immunomodulatory effects of certain antipsychotics (e.g., olanzapine, risperidone, and quetiapine) in decreasing pro-inflammatory cytokines	[311, 316]
IL-6	Predictive marker: elevated levels associated with worse response to serotonergic agents; better response to SNRIs, dopaminergic or glutamaergic modulators, and complementary anti-inflammatory agents	[311, 314, 319, 321, 323, 326, 400]
	Relationship between inflammation pre-psychotherapy and poorer response to therapy	
	Reduction after CBT in MDD patients and mindfulness	
	Linked to worse response in BD patients to ECT	
	Prognostic value: associated with improvement in depressive symptoms	
IL-10	Predictive marker: higher expression associated with better response to certain antipsychotics and antidepressants, indicating immunomodulatory effects	[315, 316 ]
IFN-γ	Predictive marker: higher levels linked to poorer response to antidepressants; reduction associated with certain antipsychotics	[311]
TNF-α	Predictive marker: modulation associated with lithium therapy, affecting various inflammatory pathways	[319, 326]
	Decrease in salivary levels after mindfulness in affective disorders; associated with poorer responses in BD patients to ECT	
CRP	Prognostic marker: elevated levels before starting antidepressant therapy associated with a worse response to serotonergic agents	[314, 325, 388]
GSK-3β	Prognostic marker: suppression linked with lithium therapy, impacting inflammatory pathways	[319]
STAT	Prognostic marker: suppression associated with lithium therapy, influencing inflammatory pathways	[319]

*IL-1β* interleukin 1 beta, *IL-4* interleukin 4, *IL-6* interleukin 6, *SNRIs* selective serotonin and norepinephrine reuptake inhibitors, *CBT* cognitive behavioral therapy, *MDD* major depressive disorder, *BD* bipolar disorder, ECT electroconvulsive therapy, *IL-10* interleukin 10, *IFN-γ* interferon-γ, *TNF-α* tumor necrosis factor-α, *CRP* C-reactive protein, *GSK-3β* glycogen synthase kinase 3 beta, *STAT* signal transducer and activator of transcription

## Conclusions

The dysfunction of the immune system in its two main aspects (neuroinflammation and systemic inflammation) represents a key etiopathogenic mechanism in the onset and development of affective disorders. However, these alterations cannot be understood in isolation, but rather as part of a complex picture in which different factors and systems interact with each other. The PNIE is the area responsible for studying the relationship between these elements and the impact of body-mind integration, placing the immune system as part of a whole. The relevance of considering the human being in a holistic way helps to understand the pathogenic complexity of affective disorders and how many of the mechanisms described mutually influence each other, enhancing and exacerbating each other. Thus, the dysfunction of the immune system can influence and activate different mechanisms that promote a disruption of the psyche, damage to the nervous system, alterations to the endocrine and metabolic systems, and disruption of the microbiota and intestinal ecosystem, as well as of other organs. In turn, all these mechanisms are responsible for inducing and potentiating the dysfunction of the immune system, thus relating all these mechanisms with the development of affective disorders. Lifestyle also represents a key element that must be considered to understand the role of immune system dysfunction in the context of PNIE, since malnutrition, a sedentary lifestyle, sleep disturbances, lack of exposure to the sun or nature and a poor quality of social relationships promote negative changes in the immune system that promote the development of affective disorders.

In fact, the clinical approach to these patients is usually multidisciplinary, and the therapeutic arsenal includes different pharmacological (i.e., antidepressants, antipsychotics, and lithium) and non-pharmacological (i.e., psychotherapy, lifestyle interventions, and ECT) treatments. These types of approaches are capable of modulating the immune system and other elements of PNIE in these patients, especially when several therapies are combined. Going deeper into the study of the immunomodulatory effect of these interventions is also a line of research of great interest due to their potential uses as predictive biomarkers or as therapeutic targets.

## Data Availability

Not applicable.

## Abbreviations

ACTH: Adrenocorticotropic hormone
APC: Antigen-presenting cell
ANS: Autonomic nervous system
BAG-1: BCL2-associated athanogene 1
BBB: Blood–brain barrier
BD: Bipolar disorder
BDNF: Brain-derived neurotrophic factor
CBT: Cognitive behavioral therapy
CNS: Central nervous system
CRH: Corticotropin release hormone
CRP: C-reactive protein
CTLs: Cytotoxic T lymphocytes
COX-2: Cyclooxygenase 2
DAMPs: Damage-associated molecular patterns
DHA: Docosahexaenoic acid
T2DM: Type 2 diabetes mellitus
DSM-5: Diagnostic and Statistical Manual of Mental Disorders, Fifth Edition
ECT: Electroconvulsive therapy
ELS: Early-life stress
EPA: Eicosapentaenoic acid
FMT: Fecal microbiota transplantation
GALT: Gut-associated lymphoid tissue
GH: Growth hormone
GHRH: Growth hormone-releasing factor
GR: Glucocorticoid receptors
GSK-3β: Glycogen synthase kinase 3 beta
HDL: High-density lipoprotein
HPA: Hypothalamic–pituitary–adrenal
HPGn: Hypothalamic–pituitary–gonadal
HPS: Hypothalamic–pituitary–somatotropic
HPT: Hypothalamic–pituitary–thyroid
IFN-γ: Interferon-γ
IGF-1: Insulin-like growth factor-1
IL: Interleukin
Kyn: Kynurenine
LBP: Lipopolysaccharide-binding protein
MDD: Major depressive disorder
NAC: N-acetylcysteine
NK: Natural killer
PAMPs: Pathogen-associated molecular patterns
PMDD: Premenstrual dysphoric disorder
PNIE: Psychoneuroimmunoendocrinology
PPD: Postpartum depression
QA: Quinolic acid
SAD: Seasonal affective disorder
SCFAs: Short-chain fatty acids
SNRIs: Selective serotonin and norepinephrine reuptake inhibitors
SNS: Sympathetic nervous system
SSRIs: Selective serotonin reuptake inhibitors
Th: T helper cells
TNF-α: Tumor necrosis factor-α
Tregs: Regulatory T cells
TRH: Thyrotropin-releasing hormone
TSH: Thyrotropin
ꞷ-3 PUFA: Omega-3 polyunsaturated fatty acid
